# Pathways to leadership: what accounts for women’s (in)equitable career paths in the health sectors in India and Kenya? A scoping review

**DOI:** 10.1136/bmjgh-2023-014745

**Published:** 2024-07-17

**Authors:** Naomi M Saville, Radhika Uppal, Sally Atieno Odunga, Sapna Kedia, Henry Owoko Odero, Sonja Tanaka, Sylvia Kiwuwa-Muyingo, Lawrence Eleh, Sucharitha Venkatesh, Zahra Zeinali, Aaron Koay, Kent Buse, Ravi Verma, Sarah Hawkes

**Affiliations:** 1 Institute for Global Health, University College London, London, UK; 2 Global Health 50/50, Cambridge, UK; 3 ICRW, New Delhi, India; 4 APHRC, Nairobi, Kenya

**Keywords:** Health policy, Decision Making, Health systems, India, Kenya

## Abstract

**Objectives:**

We aimed to capture evidence on enablers and barriers to improving equal opportunity and effective organisational interventions that can advance women’s leadership in India and Kenya’s health sectors.

**Methods:**

We systematically searched JSTOR, PubMed, SCOPUS and Web of Science databases, reference lists of selected articles and Google Scholar using string searches. We included studies that were published in English from 2000 to 2022 in peer-reviewed journals or grey literature, focused on paid, formal health professionals in India or Kenya, described factors relating to women’s representation/leadership.

**Results:**

We identified 26 studies, 15 from India and 11 from Kenya. From each country, seven studies focused on nursing. Participants included women and men health sector workers. Seven studies used mixed methods, 11 were qualitative, 5 were quantitative and 3 were commentaries. Factors influencing women’s career progression at individual/interpersonal levels included family support, personal attributes (knowledge/skills) and material resources. Factors at the organisational level included capacity strengthening, networking, organisational policies, gender quotas, work culture and relationships, flexibility, and work burden. Nursing studies identified verbal/sexual harassment and professional hierarchies as barriers to career progression. Structural barriers included a lack of infrastructure (training institutes and acceptable working environments). Normative themes included occupational segregation by gender (particularly in nursing), unpaid care work burden for women and gender norms.

Studies of interventions to improve women’s career progression and sex-disaggregated workforce data in India or Kenya were limited, especially on leadership within career pathways. The evidence focuses on enablers and barriers at work, rather than on organisations/systems to support women’s leadership or address gender norms.

**Conclusions:**

Women in India and Kenya’s health sectors face multiple impediments in their careers, which impact their advancement to leadership. This calls for gender-transformative interventions to tackle discrimination/harassment, provide targeted training/mentorship, better parental leave/benefits, flexible/remote working, family/coworker support and equal-opportunity policies/legislation.

**WHAT IS ALREADY KNOWN ON THIS TOPIC**

- The under-representation of women in leadership in the health workforce is well recognised globally, but there is a lack of data on the extent of the disparities within career pathways and the factors affecting women’s progression towards leadership, especially in low-income and middle-income countries. The career trajectory of women in the health workforce is shaped by prevailing gender norms operating at the macrolevel, as well as structural, organisational and individual factors.

**WHAT THIS STUDY ADDS**

- Our study demonstrates that women in the health workforce in India and Kenya face multiple barriers to career advancement, which are affected by harmful gender norms that burden women with unpaid care work and discrimination and potentially prioritise and normalise men’s careers and leadership.
- Individual/interpersonal enablers to career progression were family support and personal attributes while organisational enablers included a supportive workplace culture with flexible working and maternity benefits, capacity building and mentorship opportunities, and gender equality policies.

**HOW THIS STUDY MIGHT AFFECT RESEARCH, PRACTICE OR POLICY**

- Laws and policies against gender-based discrimination and harassment and to improve parental benefits and flexible working must be developed and enforced. Infrastructure, such as safe public transportation and workspaces, should be developed to retain women in their jobs. Interventions are needed to address unequal gender norms and improve support, mentorship and capacity building for leadership.

## Introduction

Under-representation of women in leadership in the health sector globally^1^ restricts women’s contributions to the sector and progression towards gender equality goals.^2^ Women comprise ~70% of those working in health but hold only 25% of senior roles.^3^ There is global commitment to women’s ‘full and effective participation and equal opportunities for leadership’ (Sustainable Development Goal(SDG) 5.5), and inclusivity and diversity in leadership are associated with positive outcomes in organisational performance in many sectors.^4 5^ In pursuit of gender justice, it is critical to address gendered leadership imbalances within the health sector workforce.

Health sectors are complex ecosystems where power and privilege are exerted including through hierarchies of professions. Many countries’ health workforces are subject to distinct gender divisions. Patriarchal norms have dictated that higher status, higher-paying professions such as medicine and dentistry be dominated by men and nursing, caregiving or support roles be performed by women.^6^ Such norms are reinforced by wider political and socioeconomic contexts and strategies of occupational closure, as explored further by Gideon *et al* in this collection^7^ and demand feminist leadership to tackle the inequalities that often characterise employment in the health sector, as argued by Hawkes and Baru.^8^

Career pathways in the health sector vary by professional cadre—including medical and nursing professions, professions allied to health and medicine (eg, occupational therapy, physiotherapy), laboratory and scientific service delivery, research staff, administration and so on. To design strategies for improving gender equity across this complex workforce and enable women to reach leadership levels, evidence is required to identify opportunities for action. In this scoping review, we synthesise evidence to describe the nature and extent of under-representation of women in leadership and the barriers and enablers for advancing equal opportunities in the health sectors. We focus on India and Kenya as large lower-middle-income countries with complex health systems and as regional hubs with a diverse range of health actors, organisations and sectors. India and Kenya are also the focus countries for the study’s funder on women in leadership in the health sector. An accompanying paper reviews existing legal provisions to support gender equality in the workplace in these countries^9^ while Buse *et al* explore the role that accountability could better play in promoting equal opportunities to formal leadership, including in the health sector.^10^

### Gender inequality in the health sector workforces in India and Kenya

**Figure 1:**
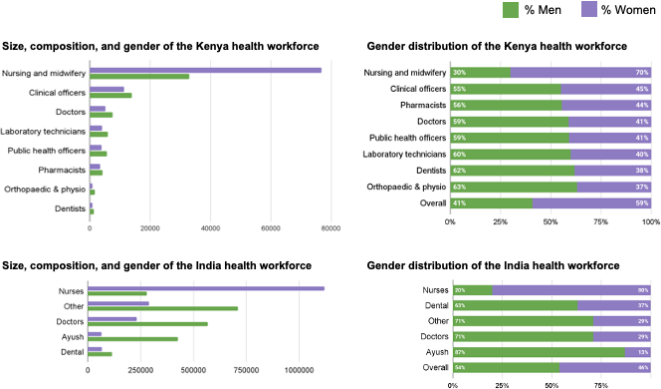
Size, composition and gender distribution of the health workforce in India and Kenya. Data are extracted from Karan *et al* for India^14^ Okoroafor *et al* for Kenya.^15^

While evidence on the representation of women in health is growing at the global level,^1 3 11–13^ evidence is lacking on gender equality in the health workforce in low-income and middle-income countries, on context-specific barriers to women’s career pathways, particularly for women who experience multiple forms of discrimination, and on effective strategies to promote women’s advancement into positions of leadership. Publicly available data on human resources for health in India and Kenya are also inadequate to arrive at a reliable estimate of size, composition and/or distribution of the health workforce and details on gender distribution across levels and categories are unavailable. Figure 1 provides available sex-disaggregated data on the health workforce in India^14^ and Kenya.^15^

The lack of regularly updated human resource data on the health workforce in India means that estimates on its size and distribution have been amassed from multiple sources including the 2001 census and National Sample Survey Office (NSSO) surveillance. Women accounted for 37% of dentists, 29% of doctors and 13% of traditional and non-conventional healthcare practitioners,^14^ but 80% of nurses and midwives. It is unclear from the data which professional cadres reach leadership roles, nor what the distribution of leadership by gender looks like.

Disaggregated data on the health workforce in Kenya are also scarce. In 2020, 58% of the 189 932 health workers in Kenya were women, across 13 major health occupations in the public and private sectors.^15^ While 70% of nurses and midwives were women, less than half of health workers were women in all other categories. The data reflect different career paths for women and men. The only occupation where women outnumber men is nursing/midwifery, which is relatively low-paid and deemed ‘low-status’ by many as compared with other health occupations.^14 15^

In India and Kenya, available data are insufficient to inform an understanding of the extent of inequality in career pathways and leadership opportunities in the health sectors. The lack of grading by sex/gender within a health occupation renders invisible any inequalities in the gender distribution of senior positions or career pathway progression. Further, published national-level health workforce data are not disaggregated by demographic characteristics beyond sex/gender within broad occupations, specialisation, and, in some cases, age, thus prohibiting an exploration and understanding of the level of representation of people and communities that face discrimination and marginalisation. Similarly, an analysis of trends in the gender distribution within occupations over time is not possible.

### Aim

This scoping review aimed to (1) map the available literature on women’s career pathways in the health sector in India and Kenya; (2) identify gaps in existing knowledge and (3) synthesise the evidence on enablers and barriers to equal opportunity and effective organisational interventions that can advance women’s leadership in the health sectors of India and Kenya.

## Methods

### Protocol

We anticipated the literature on women’s career pathways in the health system in India and Kenya would be both very heterogeneous in nature and limited in quantity. To identify the types of evidence available and understand knowledge gaps, a scoping review method was selected as the most appropriate approach.^16^ This review is reported in line with the Preferred Reporting Items for Systematic Reviews and Meta-Analyses Extension for Scoping Reviews (PRISMA-ScR)^17^ (see online supplemental appendix 1 for the PRISMA-ScR checklist) and is registered at https://osf.io/72pw9/.^18^

10.1136/bmjgh-2023-014745.supp1 Supplementary data

### Search strategy and eligibility criteria

Our search strategy (online supplemental appendix 2) was constructed based on the population, exposure, context and outcome (PECO) framework^19^ (table 1) and informed by Mousa *et al*’s systematic global review on organisational best practices for advancing women in leadership.^12^ It was further developed with an experienced librarian at Boston University and refined in an iterative process in regular meetings of the review team. We searched JSTOR, PubMed, SCOPUS and Web of Science due to their international coverage of health research. Articles in English that were published between 1 January 2000 and 30 October 2022 were included.

10.1136/bmjgh-2023-014745.supp2 Supplementary data

**Table 1:** Inclusion and exclusion criteria

PECO component	Inclusion criteria	Exclusion criteria
Population	Women health professionals in India or Kenya Publishes findings disaggregated by sex (unless they focused on the nursing sector) and by country for India or Kenya	Non-health professionals, informal health workers, community volunteers Health professionals outside of India or Kenya No disaggregated data by sex (unless they focused on the nursing sector) and by country for India or Kenya
Exposure	Barriers or enablers to advancing women’s leadership in the health sector; interventions to advance women’s careers in the health sector	No description of women’s representation or leadership in the health sector or does not focus on nursing careers
Context	Any of the WHO health system building blocks^57^ in India and Kenya, including leadership, health workforce and training and service delivery	Outside of the health system
Outcome	Career advancement or career stagnation and attrition	Not related to career retention or advancement

We set 2000 as our earliest date as this was when the Millennium Development Goals shifted the global agenda for gender equality. Only studies published in the English language were included as it is the primary academic publishing language in India and Kenya, and the shared language of the review team. Review-type articles, for example, systematic reviews were excluded, but they were screened for relevant studies.

Other relevant literature was identified through searches of Google Scholar. The additional value of Google Scholar to searching academic databases is well established, although searches are not replicable.^20^ Given the small number of studies identified through bibliographic databases, we supplemented database searches with free-text searches of grey and peer-reviewed literature in Google Scholar using the terms: “Gender” AND “women” AND “career” AND “health sector OR health system OR health worker OR health workforce” AND “Kenya OR India”, rendering eight different combinations. The first 400 results of each Google Scholar search were screened.^20^

Additionally, the reference lists of relevant studies were handsearched for more references. The final search results were exported into Microsoft Excel, and duplicates were removed by authors ZZ and LE.

We included studies that examined nursing career pathways and leadership regardless of whether the data or findings were sex disaggregated. Nursing is a women-dominated profession, a function of occupational segregation driven by gender stereotypes. Given that such professions tend to be afforded lower social value, pay and prestige, and offer fewer opportunities to advance into positions of leadership, we included all studies on nursing careers to account for nursing-specific gendered career barriers for women and men.

### Selection of sources of evidence

Study screening was conducted on Microsoft Excel. Three reviewers (ZZ, ST and SH) conducted a pilot text and abstract screening of the same 100 publications, discussed the results and amended the eligibility criteria. Two reviewers then conducted a title and abstract review of all search results (ZZ and LE). Nine authors (NMS, RU, SAO, SK, ST, SK-M and SH) who composed the main review team discussed the initial results, disagreements between reviewers and agreed on the publications for full-text review.

Subsequently, six reviewers screened the full text of all selected publications (SH, RU, SV, HOO, SAO and NMS). Two reviewers screened each publication and conflicts were resolved by team discussion and consensus.

## Data extraction

A data extraction form was developed by the review team in Microsoft Excel to determine which variables to chart (see online supplemental appendix 3). Five reviewers piloted the data extraction form by reviewing two publications selected following full-text screening (NMS, SH, ST, SK and SAO). The review team discussed these results and amended the data extraction form. Data extraction was conducted by three reviewers for studies on India (SK, RU and SV) and three reviewers for studies on Kenya (SAO, HOO and LE). At least two reviewers conducted data extraction and compared results for each publication. Inconsistencies in data extraction were resolved by consensus and discussion with other reviewers if needed. Where relevant evidence for data charting was found that referenced another article, we went to the original article to assess its eligibility for inclusion and charted data from the original source wherever applicable. We ensured that data from the same article had not been charted more than once.

10.1136/bmjgh-2023-014745.supp3 Supplementary data

The data extraction form captured relevant information on key study characteristics and findings, including authors; publication year; methods; research question; demographic and professional characteristics of the study population; disaggregated measures of career pathways; enablers and barriers to improving equality and inclusive leadership for women in the health sector; description and evidence of effectiveness of intervention and reviewers’ comments on findings or quality of the publication. No appraisal of the risk of bias or methodological quality was conducted, as this is neither within the scope of this work nor generally expected of scoping review methodology.^21^

### Synthesis of results

We compiled and summarised the characteristics of the included studies. To synthesise the study findings, we used the socioecological model. We selected the model as it considers factors at individual, organisational, community and societal levels, focuses on the continuous interaction between the person and their environment, and acknowledges intersecting identities and experiences.^22 23^ We use this model in recognition that efforts to achieve gender equality in the workplace are situated within complex systems and that contextual factors may moderate intervention efforts aimed at the individual level.

Using qualitative content analysis,^24^ pairs of reviewers for each country (India: SK, RU, SV; Kenya: SAO, HOO) deductively coded study findings into different levels of the model by extracting text from the charted data and allocating it to common themes which emerged across studies and contexts. For enablers and barriers to women’s career progression in the health sectors, the themes were iteratively discussed by the review team. For interventions to improve women’s career progression we compiled a narrative summary of findings as the studies were too few to collate and analyse thematic content.

### Patient and public involvement

As this is a review of completed studies, we did not engage patients or the public.

## Results

### Study characteristics

For India-related studies, after excluding duplicates, 2100 studies were identified from searches of electronic databases, 33 from Google Scholar and 6 from reference searching. Based on the title and the abstract, 2100 were excluded, with 49 full-text articles retrieved and assessed for eligibility. Of these, 15 studies were considered eligible for this review.

For Kenya-related studies, after excluding duplicates, 3304 studies were identified from searches of electronic databases, 17 from Google Scholar and 2 from reference searching. Based on the title and the abstract, 3275 were excluded, with 29 full-text articles retrieved and assessed for eligibility. Of these, 11 studies were considered eligible for this review.

Out of the total 26 studies included, 7 nursing-specific studies were included in each final sample for India and Kenya.

**Figure 2:**
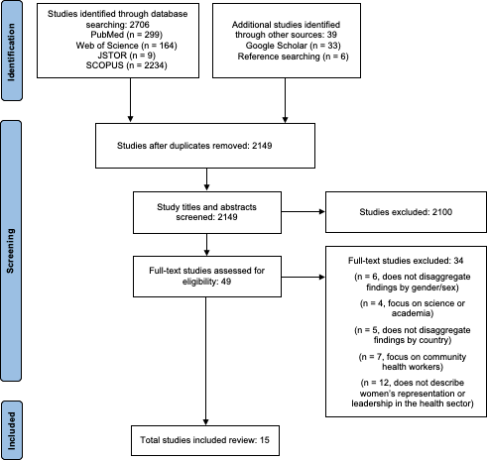
Flow diagram of selection of studies included in the review, India.

**Figure 3:**
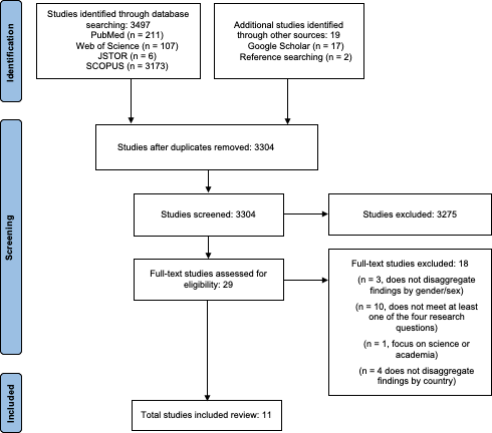
Flow diagram of selection of studies included in the review, Kenya.

Figures 2 and 3 provide PRISMA flow diagrams for India and Kenya, respectively.

Characteristics of the 26 studies included in our review are presented in table 2. Of the included studies, 11 (42%) related to Kenya (publication year 2007–2022 with data collection period ranging from 2008 to 2020) and 15 (58%) to India (publication year 2013–2022 with data collection period ranging from historical texts dating back to 1892–2019). Most of the studies, that is, 24 (92%), were descriptive. Seven (27%) studies employed mixed methods, 11 (42%) were qualitative, 5 (19%) were quantitative and 3 (12%) were commentaries. Samples included diverse populations across different career stages, positions and parts of the health sector, but mostly focused on professionals engaged in healthcare service delivery. Only one in each country described an intervention to advance women’s careers.

**Table 2:** Summary of included studies

	India					
SN	Author and year	Population and number of participants	Context and location	Study period	Focus of study	Type and methods employed
1	Bhadra (2011)^45^	Women in medical professions (n=NA)	Profession of medicine, India	1880–2010	Women’s participation in the medical profession	Historical commentary, descriptive (women’s historical participation as doctors and gender issues in medicine)
2	Chopra (2018)^35^	Women oncologists (n=171)	Academic institutes and private practice, India	2018	Work-related challenges and performance improvement	Quantitative (online survey modelled on European Society of Medical Oncology survey), descriptive (gender disparities, pay gap and performance)
3	Nair (2017)^43^	Male and female ophthalmologists (n=297; 101 women, 196 men)	Public hospitals and private practice in Mumbai, India	2015	Work-related challenges and work satisfaction	Quantitative (survey), statistical analysis using GraphPad Prism 6, descriptive (differences between experiences of male and female ophthalmologists)
4	Nambiar (2022)^25^	Women leaders in health (n=15)	Public health sector, civil society, private practice, Kerala, India	2022	Leadership-related challenges and facilitators	Qualitative (interviews), descriptive (health workforce participation, historical context for women’s leadership in health and lessons learnt)
5	Palanisamy (2019)^26^	Women neurosurgeons (n=55)	Members of the Neurological Society of India (across sectors)	2017	Work-related challenges and motivation	Quantitative (survey), descriptive (gender disparities, support at work and work satisfaction)
6	Sood (2010)^37^	Women physicians (n=NA)	Academic, public and private practice, India	2008	Women’s participation in the medical profession and related challenges	Commentary drawing on data on the Indian health workforce, descriptive (gender disparities, occupational segregation, different working styles, harassment and abuse, mentorship and training)
7	Tandon (2007)^40^	Women in dental academia (n=1063 women dental teachers)	Public and private dental colleges, India	2007	Leadership-related challenges	Quantitative (cross-sectional survey), descriptive (gender disparities in caregiving, leadership styles and role of men)
8	Zodpey (2021)^46^	Health sector workforce (n=NA)	Public and private health sectors, India	2017–2019	Workforce data analysis	Epidemiological/surveillance (quantitative using data from National Health Workforce Accounts, Global Health Workforce Statistics, Periodic Labour Force Survey, National Sample Survey Office and Rural Health Statistics), descriptive (size and composition of health workforce, skill-mix, vacancy rates and policy implications)
	Nursing-specific studies from India					
9	Das (2021)^44^	Nurses and midwives (n=NA)	Public primary healthcare facilities, India	2021	Work and knowledge-related challenges	Qualitative (ethnographic), descriptive (knowledge, hierarchy, gender bias and devaluation of care work
10	Lakshmi (2012)^27^	Women nurses (n=200)	Government and private hospitals, Chennai, Tamil Nadu, India	2012	Work-related challenges and facilitators and work satisfaction	Qualitative (survey and secondary research), descriptive (comparison between female nurses in government and private hospitals related to work–life balance)
11	Marath (2015)^47^	Women head nurses (n=30)	Private clinical setting (hospitals), Ernakulam, Kerala, India	2015	Leadership training intervention	Mixed methods (quantitative survey based on a quasi-experimental, pretest multiple post-test control group design, focus group discussions), interventional evaluation based on Systems Research Organising Model
12	Mayra (2021)^30^	Women and men nurses and midwives in senior roles (n=34 nursing and midwifery leaders; n=8 international experts)	Public and private administration, Rajasthan, Odisha, Bihar, Madhya Pradesh, West Bengal and national level, India	2018–2019	Regulatory framework of nursing and midwifery	Qualitative (interviews), descriptive (review of frameworks and governing bodies, challenges and weaknesses, gender disparities and recommendations)
13	Nair (2016)^41^	Nursing profession (n=150 nurses)	Nursing sector, India	2016	Work-related challenges	Qualitative (interviews), descriptive (occupational segregation, status of nursing and role of religion, caste, and class)
14	Raha (2009)^38^	Nursing and midwifery profession (n=NA)	Nursing sector, Uttar Pradesh and Tamil Nadu, India	2009	Work-related challenges	Review, descriptive (quality of training, career advancement, institutional reform, policy implications and recommendations)
15	Varghese (2018)^32^	Government officials, researchers, nursing associations and nurse educators (n=9)	Public and private nursing sector, India	2013	Nursing leadership-related challenges	Mixed methods (policy review, expert interviews), descriptive (policy reforms and institutional strengthening)

	Kenya					
	Author and year	Population and number of participants	Context and location	Study period	Focus of study	Type and methods employed
1	Daniels (2013)^31^	Early and mid-career women medical doctors (n=12)	Research training programmes at US-based university	2008–2010	Training intervention and training motivation	Qualitative (interviews), an evaluation of an intervention (an international training and research programme)
2	Dhatt (2017)^29^	Women and men health workers in Kenya, Zimbabwe and Cambodia (n=64)	Mombasa and Kilifi counties in Kenya; Kwekwe, Chirumanzu, Gokwe North and Gokwe South districts in Zimbabwe; Battambang and Moung Russei districts in Cambodia	2016	Leadership-related facilitators and barriers	Mixed methods (quantitative (analysis of secondary data), qualitative (interviews), literature review)), descriptive (gender equity, women’s leadership and health systems strengthening)
3	Muraya (2019)^28^	Women and men healthcare leaders (n=25)	County-level (Mombasa and Kilifi) public and private healthcare facilities	2015–2016	Leadership-related facilitators and challenges	Qualitative (interviews), descriptive (career trajectories and experiences of healthcare leaders and the role of gender on career progression)
4	Muraya (2022)^58^	Women and men healthcare leaders (number is not specified)	County-level (Mombasa and Kilifi) public and private healthcare facilities	Not specified	Leadership-related facilitators and challenges	Qualitative (interviews), descriptive (gender quotas and health Leadership in Kenya)
	Nursing-specific studies from Kenya					
5	Appiagyei (2014)^36^	Administrators and students of nursing training institutions (n=9 administrators; number of students not specified)	Public, private and faith-based nursing training institutions in Kenya	2010	Training-related challenges	Mixed methods (quantitative analysis of secondary data and interviews), descriptive (factors affecting preservice training capacity and production of Kenyan nursing workforce)
6	East (2014)^39^	Senior women nurses (n=10)	Public, private and not-for-profit institutions in Kenya	Not specified	Work-related opportunities and challenges	Qualitative (interviews), descriptive (potential for advanced nursing practice role development in Kenya)
7	Juma (2014)^33^	National decision-makers and nurse leaders, provincial managers, and front-line nurse managers (n=32)	Public sector in two provinces (Nyanza and Central)	Not specified	Work-related opportunities and challenges	Qualitative (interviews), descriptive (nurses’ involvement in national policy development processes)
8	Mbuthia (2021)^59^	Mid-level and senior nurses (n=47)	County-level public hospitals	2019–2020	Work-related challenges	Qualitative (interviews), descriptive (new nurses professional identity transitions, violations and reconciliations)
9	Ojakaa (2014)^42^	Nurses, clinical officers and other healthcare workers (n=404)	Public and private primary healthcare facilities	2011	Work-related opportunities and challenges	Mixed methods (surveys, interviews), descriptive
10	Shariff (2014)^34^	Nurse leaders working in national or provincial leadership positions (n=78)	Health policy development in Kenya, Uganda and Tanzania	2009–2010	Work-related opportunities and challenges	Mixed methods (Delphi survey with qualitative and quantitative components), descriptive (facilitators and barriers to nurse leaders’ participation in health policy development)
11	Shariff (2015)^60^	Nurse leaders working in national or provincial leadership positions (n=78)	Health policy development in Kenya, Uganda and Tanzania	2009–2010	Leadership-related facilitators	Same dataset as Shariff^34^

NA, not available.

### Evidence of enabling factors or barriers

**Figure 4:**
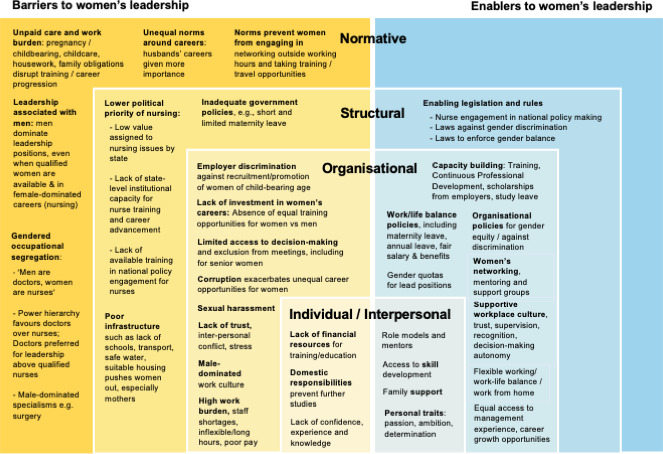
Facilitators and barriers to women’s career progression in the health workforce of India and Kenya at different levels of the socioecological model.

14 themes of facilitators and barriers were identified across the two countries at individual/interpersonal, organisational, structural and cross-cutting normative levels. These are captured in table 3 and summarised in the adapted socioecological model in figure 4.

**Table 3:** Findings on enablers and barriers to women’s equality of opportunity in health sector careers in India and Kenya

Theme	Facilitators and barriers in India	Facilitators and barriers in Kenya
Socioecological model Level 1. Individual and interpersonal		
1.1 Family support	Facilitators: Family support and encouragement including supportive natal and marital family^25 26^	Facilitators: Family support and encouragement including supportive parents and spouses^28^
1.2 Knowledge/ skills and personal attributes	Facilitators: Driven by ‘social commitment’ roles at the community level^25^ Having passion to work in a specialisation such as neurosurgery^26^ Advantage of women’s ability to multitask^25^ Barriers: Feeling of dependence on men and lack of confidence ‘*As women, we are ruled by our father, brother, husband and son at different stages of our life… It is our lack of confidence and attitude that only if men are there will we succeed. There is a dependence… we surrender too easily*.’^30^ #	Facilitators: Developing professional traits for career advancement including self-determination, discipline, passion, proactiveness, strategic thinking, ambition^28 33^ # Developing professional skills to participate in health policy-making, including leadership, policy development, negotiation, networking, problem solving and management skills, and diversification of skills outside of traditional nursing^33 34 60^ # Having role models and personal mentors^28 31^ Appealing to women’s personal interests, work environment preferences and personal and career aspirations^31^ # Barriers: Lack of confidence and lack of active participation when involved in policy-making as detriments to career progression^34^ # Lack of experience and knowledge pertaining to health policy development processes and lack of tertiary education contributes to the inability to participate in health policy development^34^ #
1.3 Material resources		Barriers: Lack of financial resources for additional education and training, especially for parents^28^
Socioecological model level 2. Organisational		
2.1 Capacity building and networking	Facilitators: Developing professional performance through short-term intensive fellowship and mentorship programmes^35^ Exclusive women’ s forum and social media groups are beneficial 26 Mentorship, career opportunities and trainings critical for workplace satisfaction^27^ # Barriers: Absence of equal overseas training opportunities for women compared with men colleagues was reported by 17% respondents of women oncologists. Many felt that inability to attend professional advancement courses affected their professional development^35^ Networking post working hours is challenging for women^35^	Facilitators Engagement of staff in Continuous Professional Development programmes such as training on different skills including health policy development^28 33 36^ # Provision of study scholarships from employers to encourage advancement of studies^28^ Broadening of the curriculum coverage in order to understand broader policy-related issues^33 34^ # Availability of professional mentors and role models for career support and sharing experiences^31 34 36^ # Having supportive supervisors to encourage career progression^28^ Barriers Inadequate institutional/financial resources to sponsor staff for Continuous Professional Development^36^ # Failure of the curriculum to cover content related to health policy-making processes^33^ #
2.2 Career progression and leadership opportunities	Facilitators: Need to have dedicated seats for women in leadership positions^35^ Women leaders have higher representation of women within the department^35 37^ and have higher proportion of women at senior ranks^35^ Barriers: Easier for men colleagues to get leadership opportunities in multilaterals organisations and abroad due to increased likelihood of being in a position to leave family to pursue such opportunities^25^ Limited possibility for career advancement for nurses in public health nursing^38^ # Corruption has to be tolerated because nurses who speak up lose their jobs and those who collude with it are promoted^30^ #	Facilitators Nurses and other healthcare workers remain in their workplaces because of availability of promotion opportunities^42^ #
2.3 Organisational policies	Facilitators: Instituting gender equity and/or anti-gender discrimination policies^35^ Organisational support for instance in the form of maternity leave benefit impacts satisfaction at workplace^27^ #	Barriers Policy challenges inhibit nurses to become in-charges^39^ #
2.4 Work culture and relationships	Facilitators: Women had supportive men and women colleagues and support from their department^26^ Cordial work environment and recognition by colleagues and seniors affects workplace satisfaction^27^ # Barriers: Gaining professional trust of colleagues and seniors a challenge which also impacts referral rate among early-career women doctors^35^ Presence of men dominated work culture, that is, challenges in working with men supervisors and colleagues,^25^ insecurity among men colleagues if women do better^40^ Increased stress among nurses due to interpersonal conflicts at the workplace^27^ # Prevalence of harassment including sexual harassment in the workplace among nurses^41^ #	Facilitators Existence of some level of decision-making autonomy in management of work^39^ # Creation of career growth opportunities including being allowed to participate in policy-making processes in all the stages^33 34^ # Nurses and other healthcare workers remain in their workplaces because of supervisor support, provision of annual leave, good salary and benefits^42^ # Barriers Limited decision-making autonomy despite being in a senior position and having significant experience^39^ # Difficulty engaging in health policy development process because of lack of opportunity/forums, lack of support from the institutions, lack of recognition of efforts, inadequate involvement and uneven participation in policy meetings^33 34^ # Nurses and other healthcare workers leave their workplaces because of inadequately supportive supervision, excessive workloads, lack of necessary equipment, job insecurity and insufficient payment of support staff^42^ # Employer discrimination against women of childbearing age at the hiring stage^29^
2.5 Flexibility and work burden	Facilitators: Flexibility in working hours and workplace such as work from home^35^ and good work–life balance important for workplace satisfaction^27^ # Barriers: Long and/or inflexible working hours^25 43^ Increased work burden due to competing priorities and staff shortage^44^ #	Facilitators Flexible work environments that enable balancing of family, career and further studies^28 31^ Availability of study leave provision to enable further studies while in-service^28^ Barriers Time limitations to engage in further studies because of the nature of the jobs^28^
Socioecological model level 3. Structural		
3.1 Governmental policies	Barriers: Short duration^40^ and limited scope^37^ of maternity benefit policy	Facilitators Inclusion of national nurse leaders in policy development, enhancing the numbers of nurses at policy development level and enforcing gender balance^34^ #
3.2 National and State institutions	Barriers: Training: Inadequate increase in number of training institutes^30^ and lack of state institutional capacity to provide additional necessary mandatory training for nurses to be eligible for promotion^38^ # Institutions: Nursing leadership dominated by men^38^; Limited participation of nurses in policy formulation and absence of nurse-specific institution at state level affecting representation of nurses issues; nursing issues not given enough importance by the State^32^ #	
3.3 Access to public infrastructure		Barriers Nurses and other healthcare workers leave their workplaces because of poor transport system to workplace, lack of access to good schooling, limited access to safe water, lack of suitable housing^42^ #
Crosscutting normative factors		
4.1 Gendered occupational segregation	Barriers: Certain specialties continue to be dominated by men such as some surgical specialties, perhaps due to long working hours.^37 45^ Men also prefer clinical work and women prefer stability of administrative work^25^ Power hierarchy between nurses and doctors, with the latter seen as superior^30 32^	Barriers Medical doctors tend to be preferentially selected for leadership positions than nurses even when nurses have relevant qualifications because of hierarchy issues^28 29 39^ #
4.2 Unpaid care work burden	Barriers: Domestic responsibilities including household work and childcare impact their career decisions^35 40 43 46^ Although 80% of women oncologists felt they had equal opportunities for training, 33% said that family responsibilities prevented them from taking the opportunities^35^	Barriers: Pregnancy, childbearing and family obligations as key factors in student, training and career disruption and attrition^28 36^ # Employers perceive women as unsuitable for managerial positions as they believe they will not be able to balance childcare and work responsibilities^29^
4.3 Norms around masculinity	Barriers Husband’s career seems to be given more importance than wife’s leading to women compromising their career development^40^	

Indicates factors relating to the nursing profession, rather than discussing women’s career progression in the health workforce overall.

#### Individual/interpersonal factors

The main individual/interpersonal factors enabling or hindering women’s career progression across both countries were family support, knowledge, skills and personal attributes, and material resources.

### Family support

In India, support from family members such as grandparents, parents, uncles and aunts inspired women to have ambitions to take up leadership positions.^25^ In an Indian survey of women neurosurgeons,^26^ 96% of participants reported receiving good to excellent support from their family members. Women nurses in Tamil Nadu reported increased work satisfaction if their childcare responsibilities were equally divided or mostly managed by their husbands.^27^

In Kenya, family support contributed to career progression. Women healthcare managers felt that they advanced in their careers because of support and encouragement from their spouses and parents.^28^ Conversely, family and parental obligations, including pregnancy and childbearing, hindered women from taking up certain job positions or furthering their education, limiting opportunities for career advancement and taking leadership positions.^28 29^

### Personal attributes

In India, women leaders draw confidence from early-career social commitment-related roles. Connection with and support from the community encouraged them to take on more responsibility.^25^ Women leaders emphasised motivation and coalition building and exercised self-awareness in crafting their roles as leaders. They saw their ability to multitask as a strength though they see this attribute as strongly gendered.^25^ Nurses mentioned a lack of confidence and dependence on men as a reason for the position of nurses diminishing and doctors not treating them as equals.^30^

In Kenya, women with the required knowledge and skills, combined with certain personal attributes, were reportedly more likely to get leadership positions and advance in their careers.^28 31^ Personal attributes mentioned by women included self-discipline, determination, passion, commitment and ambition.^28^ Personal mentors and role models also led to career growth.^28 31^

Kenyan nurse leaders and nurses said professional traits and leadership attributes such as management skills, negotiation skills, problem-solving skills and effective interpersonal skills contributed to them advancing to leadership positions.^32–34^ Nurse leaders also mentioned that being knowledgeable about health policy formulation and competent in their field of nursing contributed to them being included in policy development processes.^32 34^ Conversely, lack of confidence, lack of active participation when involved in policy-making, lack of experience and knowledge pertaining to the health policy development process, and lack of tertiary education all contribute to the inability of nurses to participate in health policy development and deter career progression.^34^

### Material resources

Financial limitations prevented some Kenyan women healthcare managers from advancing in their careers. Those with parental obligations mentioned having insufficient financial resources to further their own education and educate their children at the same time, which limited their career growth opportunities.^28^

#### Organisational factors

Organisational factors enabling or hindering women’s career progression were capacity building and networking, career progression and leadership opportunities, organisational policies, work culture and relationships, flexibility and work burden.

### Capacity building and networking

Indian women oncologists highlighted the critical role of capacity building for developing their professional performance, including short-term intensive fellowship programmes and mentorship programmes (recognised as important factors by 52% and 19% of survey participants, respectively). Although 80% said that they received equal opportunities for training, 33% were unable to use them due to personal or family reasons and 17% felt they lost overseas training opportunities to male colleagues.^35^ Among women nurses in India access to mentorship, career opportunities and training increases workplace motivation and satisfaction.^27^

In an Indian study of women neurosurgeons, (89%) found a women-only social media group beneficial and 67% felt that an exclusive women neurosurgeons forum would be very advantageous for them.^26^ In contrast, networking after office hours has been challenging for women in India.^35^

In Kenya, women’s engagement in continuous professional development programmes such as on-the-job training helped them to develop the skills and knowledge needed to advance in their careers and take up leadership positions. The provision of scholarships to further women’s studies created an opportunity for women to take up roles that contribute to career progression.^28^ Women doctors and healthcare managers reported that workplace mentors and supportive supervisors enabled them to advance in their careers.^28 31^

Kenyan nurses’ participation in continuous professional development such as training contributed to knowledge acquisition in key areas needed for career progression.^33 36^ Nurses noted that a broadened curriculum that incorporates policy-related issues enabled them to better understand the health policy development process and participate in its formulation.^33 34^ However, the lack of institutional financial resources meant Kenyan nurses could not be sponsored for continuous professional development.^36^ Moreover, the fact that curricula fail to cover content related to health policy formulation also contributed to nurses’ lack of knowledge to participate in the development of health policies.^33^ Kenyan nurse leaders said professional mentors and role models helped them understand, navigate and participate in the health policy development process by offering support and sharing their experiences with them.^34 36^

### Career progression and leadership opportunities

In India, departments headed by women have a higher proportion of women in senior positions.^35 37^ Dedicated seats for women in leadership roles and in national scientific bodies have the potential to improve work performance of women oncologists.^35^ However, challenges remain for women in taking on leadership roles, especially in multilateral organisations and out of the country, given the social expectations and family responsibilities put on women.^25^

Within the public health system, the possibility of career advancement is limited for nurses.^38^ Corruption also impacts nurses’ career growth opportunities with those who collude with it being favoured over others.^30^

### Organisational policies

In India, promotion of gender equity and/or antigender discrimination policies is critical for the workplace.^35^ Organisation-level policies such as maternity leave benefits and family allowance have a positive impact on nurses’ satisfaction.^27^ In Kenya, experienced nurses reported that a change in policy was needed before they could become in-charges who manage rural hospitals, as these roles are only open to clinical officers currently.^39^

### Work culture, relationships and harassment

In an Indian study of women neurosurgeons, most reported receiving positive support from their department during their residency, however, some reported receiving poor or no support and 40% of women reported facing gender discrimination.^26^ Gaining trust from colleagues is challenging, as 40% of Indian women oncologists stated women doctors do not get equal patient referrals, especially early in their careers.^35^ In Kenya, the perception of women as child bearers and nurturers was damaging to their career progression, with both men and women supervisors assuming that women would not show commitment once they have a child.^29^

Male-dominated work culture presents another barrier for women in India, for example, women face challenges while asking men supervisors for childcare-related leave.^25^ Men may experience insecurity if women perform better, as highlighted by a survey in which 55% of women shared that men feel uncomfortable when a woman performs better.^40^ Work satisfaction among Indian nurses increases if the work environment is cordial and they are recognised by colleagues and seniors while interpersonal conflict leads to increased stress in the workplace.^27^

In India, verbal and sexual harassment is a major impediment for nurses. For many nurses, sexual harassment is an unavoidable experience and vulnerability is higher on night shifts. The perpetrators include superiors, doctors, ward boys and other workers as well as people accompanying patients.^41^

In Kenya, nurse leaders mentioned that having some level of decision-making autonomy in their workplaces enabled them to manage their work effectively and exercise their leadership capabilities.^39^ Despite being in a senior position and having significant leadership experience, some Kenyan nurse leaders were afforded limited opportunity to make decisions independently. This constrained their ability to discharge their duties effectively as they had to consult on all decisions.^39^ Allowing nurses to participate in policy-making processes at all stages contributed to their career progression.^33 34^ Nurses and nurse leaders, however, reported difficulty in engaging in the health policy development process because of lack of opportunity or forums, support from their institutions, recognition of efforts or access to policy meetings.^33 34^

Factors related to Kenyan nurses and other healthcare workers remaining in their jobs included supervisor support, provision of annual leave, attractive salary and benefits, and availability of promotion opportunities. Reasons for leaving included inadequate supervision, insufficient pay, heavy workload, lack of job security and poor infrastructure in their workplace or location.^42^

### Flexibility and work burden

In India, flexibility is an important determinant for workplace performance and satisfaction. One-third of women oncologists highlighted flexible hours and 20% mentioned the role of digital platforms as important factors for performance at work.^35^ However, long and/or inflexible working hours pose challenges for women,^43^ often leading to work during evenings or weekends.^25^ Good work–life balance was important to work satisfaction among nurses.^27^ However, nurses often suffer a high work burden due to a shortage of clinical staff and competing priorities.^44^

In Kenya, a work environment that enables balancing of family, career and further studies enabled women’s career progression and take up of leadership positions.^28 31^ Workplaces that provided study leave enabled women to further their studies while keeping their jobs.^28^ However, time constraints to engage in further studies can limit women doctors’ or nurses’ career growth because of the nature and schedule of their jobs.^28^

### Structural factors

#### Government policies

Kenyan nurses reported that functioning legislative structures ensured a higher representation of national nurse leaders in policy development at all stages and helped to enforce gender balance.^34^ No other studies looked at the impact of structural and legal factors on women’s career progression.

#### National and state institutions

In India, insufficient numbers of nurse/midwife training institutes limited opportunities for practical experience during preservice education for nursing students in private institutions.^30^ Similarly, a lack of capacity to provide the additional training mandated for nurses in India prevented them from being eligible for promotion.^38^

#### Access to public infrastructure

In Kenya, lack of necessary equipment to do their jobs, poor transport to the workplace, lack of access to good schools for children, limited access to safe water and lack of suitable housing caused health workers to leave their jobs.^42^

### Cross-cutting gender normative factors

#### Gendered occupational segregation

In India, there are power differentials between nurses and doctors.^30^ Nurses report that nursing is perceived as an ‘unskilled’ profession. One participant stated ‘Leadership grows in social contexts and hierarchical settings, but a nurse is the lowest in this hierarchy’.^32^ Even in the women-dominated profession of nursing, women are often not part of decision-making bodies with doctors seen as superior.^30^ Similarly in Kenya, nurses reported that medical doctors and clinical officers were preferentially selected for leadership positions even when they had relevant qualifications because of hierarchy issues within the healthcare system.^28 29 39^

Gendered occupational segregation is common in the health sector in India. Due to gender stereotypes around women’s roles and abilities, women are deemed unsuitable for or are discouraged from, joining certain specialisations.^26^ 73% of women neurosurgeons faced discouragement to join the field, including from other neurosurgeons or medical professionals and/or their families. Neurosurgery was not considered a suitable field for women due to the long-working hours, long duration of residency with a meagre stipend and the risk of professional demands impacting women’s marriage prospects.^26^ Other specialties in India, such as cardiology, surgery and orthopaedic surgery, continue to be dominated by men.^37 45^

#### Unpaid care work burden

In India, domestic responsibilities, including unpaid care work and household chores, impact women’s career aspirations, growth and decisions.^35 40 43^ Two-thirds of Indian women oncologists reported ‘domestic responsibilities’ as the main challenge to professional growth.^35^ Women ophthalmologists found that ‘greater family responsibility’ was a key difficulty.^43^ Meanwhile 57% of women in academic dentistry managed domestic work alone and 63% of women were solely responsible for childcare, which affected their career development. Further, 49% of these dentists said they compromised on their work due to their family responsibilities. The authors found that women who have ‘successful’ careers had either delayed starting a family or were single.^40^ An analysis of the 2017–18 NSSO survey found that 54% of women with a degree in medicine were out of the labour force because they were engaged in household work.^46^

In Kenya, some employers discriminated against women who were pregnant or had children during the recruitment process for a managerial position because they perceived that mothers would have difficulty balancing work and family obligations and ultimately resort to attending work irregularly.^29^ Pregnancy was a key reason why Kenyan student nurses dropped out of training, which rendered them unable to secure good employment.^36^

#### Norms around masculinity

In India, husbands’ careers were given more importance than wives’, leading to women compromising their career development. 65% of academic dentists shared that the ‘secret of a happy marriage’ is a better career path for the husband. Further, 55% reported that husbands were not comfortable if their wives had better careers.^40^

### Evidence of interventions

One Indian study examined a leadership intervention. Marath and RamachandraMarath and Ramachandra (2015) tested a facilitative nurse leadership development package (LDP) with 30 head nurses in the LDP arm compared with 30 head nurses in the control.^47^ The intervention involved training on five concepts of transformational leadership. Researchers compared self-rated and observed pretest and post-test scores between arms and found that the mean self-rated post test scores of head nurses in the experimental group showed a significant increase from pretest through all the post-test time periods for all the five leadership practices. While providing evidence on how to train leaders, the effect of the training on the nurses’ career development and growth was not tested.

One Kenyan study focused on an intervention.^31^ Kenyan women doctors were engaged in an international research training programme known as the University of Washington AIDS International Training and Research Programme. The programme included short-term and long-term training in epidemiology, biostatistics, biomedical research and sociobehavioural research. The effect of the intervention on equality of opportunity and inclusive leadership was not evaluated. However, participants who completed the programme were able to: transfer their research knowledge through teaching and mentoring, venture into research careers and develop research programmes and engage in other lucrative careers.

## Discussion

### Statement of principal findings

Our study demonstrates the complexity of multifactorial determinants of equality of career opportunity and leadership in the health sectors in India and Kenya. While both countries differ in terms of culture and diversity, they share common barriers and enablers to women’s leadership at individual, organisational and structural levels and were affected by similar cross-cutting gender norms which disadvantage women. Most nurses in both countries are women and have limited access to leadership positions. Thus, addressing power imbalances between nurses and doctors is key to enabling more women to take up leadership roles. Furthermore, interventions to transform gender norms and develop/enforce gender-equitable laws and policies are crucial to enabling women to progress into leadership in the health sectors of India and Kenya.

### Strengths and weaknesses of the study

Strengths of our review include the use of structured search methods, rigorous data extraction and analysis by independent reviewers in India and Kenya following standardised scoping review methods. Weaknesses include that we did not undertake quality appraisals of included studies and did not prespecify the inclusion of studies about nursing as a ‘women’s domain’ within the health sector. The lack of quantitative measures of women’s career progression and lack of interventional studies limit what conclusions can be drawn about what needs to change and how.

Further, our use of the selected databases may have resulted in the exclusion of articles indexed in other databases. We did not include grey literature beyond what was found in Google Scholar. Given the growing interest in women’s leadership in the health sector, including in India and Kenya, this may have resulted in the exclusion of relevant grey literature. The use of English-only articles may have resulted in the exclusion of relevant articles in other languages.

Our application of the socioecological model during the analysis of data extracted from papers/studies may have been subject to inter-reviewer differences. However, repeated discussions and initial duplicate coding of a sample of studies by several team members minimised such differences.

The health sector is one of the largest sectors that employs women and historically women are predominantly employed in the nursing profession, which could bias the presentation of findings and implications for policy. We also did not include informal or voluntary health workers, whose career progression opportunities may differ from formal paid health workers.

### Strengths and weaknesses in relation to other studies

Mousa *et al*’s systematic review of organisational interventions for advancing women in leadership across multiple sectors in mostly high-income countries identified the importance of organisational leadership, commitment and accountability. Proposed interventions such as improving workplace culture, mentoring and networking, leader training and development, role-modelling, work–life integration and gender bias elimination are consistent with those emerging from our study.^12^ Workplaces reinforce patriarchal norms and discrimination against women on the grounds of their reproductive and caring responsibilities, and verbal and sexual harassment/assault is widespread.^2 3 48 49^ Internalised gender norms around employment and work roles^11 50^ and gendered attitudes towards competition, failure and risk-taking^51^ impact women’s labour market prospects with women required to demonstrate traditionally ‘male’ characteristics in order to succeed.^28^ Studies focus on women’s ‘failings’ and what women need to do rather than addressing gender relations, with no attention given to how to address deeply embedded norms around gender roles. Hence, we concur with Hay that ‘disruptive’ and transformative action is needed both within the health sector and across wider society to address gender norms and build accountable, gender-equitable societies. This includes making equal opportunities for healthcare professionals of all genders, and moving away from the concept that women should ‘care’ while men should ‘cure’.^2^

### Meaning of the study: possible explanations and implications for clinicians and policymakers

Our review highlighted an absence of evidence on ‘what works’ to achieve inclusive and equitable career progression for women in India and Kenya, and of monitoring data to indicate whether progress is being made. Our analysis of enablers and barriers to career progression shows that career equity and leadership diversity can only be realised through action across multiple intersecting domains. The relative absence of examination and evaluation of the structural environment, as compared with individual/interpersonal and organisational factors, indicates where further research is needed—particularly given the potentially high impact of laws promoting gender equality.

### Unanswered questions and future research

Gender is just one of many aspects of identity by which people working in the health sector are discriminated against. Despite the existence of protected characteristics (sex, age, ethnicity, race and disability) in the Kenyan Constitution, and protection against discrimination on the grounds of religion, race, caste, sex or place of birth in the Indian Constitution,^52^ we found no analyses of workforce data by these characteristics in the health sectors apart from one study looking at caste discrimination^53^ and no studies which examined the intersectional barriers or enablers to career progression nor how these factors shaped occupational segregation. Such studies are needed to inform efforts to level up opportunities for marginalised women.

While our study focused on the formally paid health workforce, over a million informally employed community health workers in India^54 55^ and 73% of the 89 000 Community Health Volunteers in Kenya are women.^56^ Future studies and interventions should investigate career progression opportunities for this cadre of largely low-income women volunteers and the legal/ethical implications of relying on them to maintain front-line health systems.

Ensuring equality of opportunity for career progression for women health professions will require action on the career pathways within the different professional cadres as well as negotiating the hierarchies of intercadre professions within the leadership space.

10.1136/bmjgh-2023-014745.supp4 Supplementary data

## Data Availability

All data relevant to the study are included in the article or uploaded as online supplemental information.
