# Supplementary material for: Pathways to leadership: what accounts for women’s (in)equitable career paths in the health sectors in India and Kenya? A scoping review
Source: BMJ Glob Health. 2024 Jul 17;9(7):e014745. doi: 10.1136/bmjgh-2023-014745 (PMC11261739; doi:10.1136/bmjgh-2023-014745)
Supplement: Supplementary data [file bmjgh-2023-014745supp002.pdf]

Appendix 2. Search Strategies

India-related papers		
**Search strategy**	**Data base**	**Res ults**
(("india"[MeSH Terms] OR "india"[All Fields] OR "india s"[All Fields] OR "indias"[All Fields] OR "Uttar Pradesh"[All Fields] OR "Bihar"[All Fields] OR "Indian States"[All Fields] OR "Indian Regions"[All Fields] OR "North India"[All Fields]) NOT "North America"[All Fields]) AND (("woman*"[All Fields] OR "man"[All Fields] OR ("femal"[All Fields] OR "female"[MeSH Terms] OR "female"[All Fields] OR "females"[All Fields] OR "female s"[All Fields] OR "femals"[All Fields]) OR ("male"[MeSH Terms] OR "male"[All Fields]) OR ("gender identity"[MeSH Terms] OR ("gender"[All Fields] AND "identity"[All Fields]) OR "gender identity"[All Fields] OR "gendered"[All Fields] OR "gender s"[All Fields] OR "gendering"[All Fields] OR "genderized"[All Fields] OR "genders"[All Fields] OR "sex"[MeSH Terms] OR "sex"[All Fields] OR "gender"[All Fields]) OR "gender identity"[All Fields] OR ("sex"[MeSH Terms] OR "sex"[All Fields])) AND ("hospitals"[MeSH Terms:noexp] OR ("delivery of health care"[MeSH Terms] OR ("delivery"[All Fields] AND "health"[All Fields] AND "care"[All Fields]) OR "delivery of health care"[All Fields] OR "healthcare"[All Fields] OR "healthcare s"[All Fields] OR "healthcares"[All Fields]) OR "health system"[All Fields] OR "health organization"[All Fields] OR "health organisation"[All Fields] OR "medical institutions"[All Fields] OR "primary healthcare"[All Fields] OR "medical colleges"[All Fields] OR "government hospitals"[All Fields] OR "medical research"[All Fields] OR "private hospital"[All Fields] OR "medical boards"[All Fields] OR "health workforce"[All Fields] OR "health r&d"[All Fields] OR "workplace"[MeSH Terms:noexp] OR "health associations"[All Fields] OR "health services"[All Fields] OR "human resources for health"[All Fields] OR "community health worker"[All Fields] OR ("nurse s"[All Fields] OR "nurses"[MeSH Terms] OR "nurses"[All Fields] OR "nurse"[All Fields] OR "nurses s"[All Fields]) OR ("midwife s"[All Fields] OR "midwifery"[MeSH Terms] OR "midwifery"[All Fields] OR "midwife"[All Fields] OR "midwifes"[All Fields] OR "midwifing"[All Fields]) OR ("physician s"[All Fields] OR "physicians"[MeSH Terms] OR "physicians"[All Fields] OR "physician"[All Fields] OR "physicians s"[All Fields]))) AND ("leadership"[MeSH Terms:noexp] OR "career mobility"[All Fields] OR "career progression"[All Fields] OR "career development"[All Fields] OR "career growth"[All Fields] OR "professional advancement"[All Fields] OR "career progress"[All Fields] OR "career path"[All Fields] OR "job progress"[All Fields] OR "key decision makers"[All Fields] OR "decision makers"[All Fields] OR "health policy makers"[All Fields] OR "health directorates"[All Fields] OR "diverse leadership"[All Fields] OR (("gender identity"[MeSH Terms] OR ("gender"[All Fields] AND "identity"[All Fields]) OR "gender identity"[All Fields] OR "gendered"[All Fields] OR "gender s"[All Fields] OR "gendering"[All Fields] OR "genderized"[All Fields] OR "genders"[All Fields] OR "sex"[MeSH Terms] OR "sex"[All Fields] OR "gender"[All Fields]) AND ("transformational"[All Fields] OR "transformative"[All Fields]) AND ("leadership"[MeSH Terms] OR "leadership"[All Fields] OR "leadership s"[All Fields] OR "leaderships"[All Fields])) OR "transformational leadership"[All Fields] OR "transformative leadership"[All Fields]) AND ("glass ceiling"[All Fields] OR "leadership labyrinth"[All Fields] OR "occupational segregation"[All Fields] OR "vertical segregation"[All Fields] OR ("bias"[MeSH Terms] OR "bias"[All Fields]) OR "gender bias"[All Fields] OR "stereotyping"[MeSH Terms:noexp] OR "gender stereotype"[All Fields] OR "discrimination, psychological"[MeSH Terms:noexp] OR "sexism"[MeSH Terms:noexp])	PubM ed	11
(("india"[MeSH Terms] OR "india"[All Fields] OR "india s"[All Fields] OR "indias"[All Fields] OR "Uttar Pradesh"[All Fields] OR "Bihar"[All Fields] OR "Indian States"[All Fields] OR "Indian Regions"[All Fields] OR "North India"[All Fields]) NOT "North America"[All Fields]) AND (("woman*"[All Fields] OR "man"[All Fields] OR ("femal"[All Fields] OR "female"[MeSH Terms] OR "female"[All Fields] OR "females"[All Fields] OR "female s"[All Fields] OR	PubM ed	17

"femals"[All Fields]) OR ("male"[MeSH Terms] OR "male"[All Fields]) OR ("gender identity"[MeSH Terms] OR ("gender"[All Fields] AND "identity"[All Fields]) OR "gender identity"[All Fields] OR "gendered"[All Fields] OR "gender s"[All Fields] OR "gendering"[All Fields] OR "genderized"[All Fields] OR "genders"[All Fields] OR "sex"[MeSH Terms] OR "sex"[All Fields] OR "gender"[All Fields]) OR "gender identity"[All Fields] OR ("sex"[MeSH Terms] OR "sex"[All Fields])) AND ("hospitals"[MeSH Terms:noexp] OR ("delivery of health care"[MeSH Terms] OR ("delivery"[All Fields] AND "health"[All Fields] AND "care"[All Fields]) OR "delivery of health care"[All Fields] OR "healthcare"[All Fields] OR "healthcare s"[All Fields] OR "healthcares"[All Fields]) OR "health system"[All Fields] OR "health organization"[All Fields] OR "health organisation"[All Fields] OR "medical institutions"[All Fields] OR "primary healthcare"[All Fields] OR "medical colleges"[All Fields] OR "government hospitals"[All Fields] OR "medical research"[All Fields] OR "private hospital"[All Fields] OR "medical boards"[All Fields] OR "health workforce"[All Fields] OR "health r&d"[All Fields] OR "workplace"[MeSH Terms:noexp] OR "health associations"[All Fields] OR "health services"[All Fields] OR "human resources for health"[All Fields] OR "community health worker"[All Fields] OR ("nurse s"[All Fields] OR "nurses"[MeSH Terms] OR "nurses"[All Fields] OR "nurse"[All Fields] OR "nurses s"[All Fields]) OR ("midwife s"[All Fields] OR "midwifery"[MeSH Terms] OR "midwifery"[All Fields] OR "midwife"[All Fields] OR "midwifes"[All Fields] OR "midwifing"[All Fields]) OR ("physician s"[All Fields] OR "physicians"[MeSH Terms] OR "physicians"[All Fields] OR "physician"[All Fields] OR "physicians s"[All Fields]))) AND ("glass ceiling"[All Fields] OR "leadership labyrinth"[All Fields] OR "occupational segregation"[All Fields] OR "vertical segregation"[All Fields] OR ("bias"[MeSH Terms] OR "bias"[All Fields]) OR "gender bias"[All Fields] OR "stereotyping"[MeSH Terms:noexp] OR "gender stereotype"[All Fields] OR "discrimination, psychological"[MeSH Terms:noexp] OR "sexism"[MeSH Terms:noexp]) AND ("leadership"[MeSH Terms:noexp] OR "career mobility"[All Fields] OR "career progression"[All Fields] OR "career development"[All Fields] OR "career growth"[All Fields] OR "professional advancement"[All Fields] OR "career progress"[All Fields] OR "career path"[All Fields] OR "job progress"[All Fields] OR "key decision makers"[All Fields] OR "decision makers"[All Fields] OR "health policy makers"[All Fields] OR "health directorates"[All Fields] OR "tooth attrition"[MeSH Terms:noexp] OR "retention, psychology"[MeSH Terms:noexp] OR (("career"[All Fields] OR "careers"[All Fields]) AND ("stall"[All Fields] OR "stalled"[All Fields] OR "stalling"[All Fields] OR "stallings"[All Fields] OR "stalls"[All Fields])) OR ("lack"[All Fields] AND ("promote"[All Fields] OR "promoted"[All Fields] OR "promotes"[All Fields] OR "promoting"[All Fields] OR "promotion"[All Fields] OR "promotional"[All Fields] OR "promotions"[All Fields] OR "promotive"[All Fields])) OR ("non"[All Fields] AND ("represent"[All Fields] OR "representative"[All Fields] OR "representatively"[All Fields] OR "representativeness"[All Fields] OR "representatives"[All Fields] OR "representativity"[All Fields] OR "represented"[All Fields] OR "representing"[All Fields] OR "represents"[All Fields]) AND ("leadership"[MeSH Terms] OR "leadership"[All Fields] OR "leadership s"[All Fields] OR "leaderships"[All Fields])) OR "glass ceiling"[All Fields])		
((("india"[MeSH Terms] OR "india"[All Fields] OR "india s"[All Fields] OR "indias"[All Fields] OR "Uttar Pradesh"[All Fields] OR "Bihar"[All Fields] OR "Indian States"[All Fields] OR "Indian Regions"[All Fields] OR "North India"[All Fields]) NOT "North America"[All Fields]) AND (("woman*"[All Fields] OR "man"[All Fields] OR ("femal"[All Fields] OR "female"[MeSH Terms] OR "female"[All Fields] OR "females"[All Fields] OR "female s"[All Fields] OR "femals"[All Fields]) OR ("male"[MeSH Terms] OR "male"[All Fields]) OR ("gender identity"[MeSH Terms] OR ("gender"[All Fields] AND "identity"[All Fields]) OR "gender identity"[All Fields] OR "gendered"[All Fields] OR "gender s"[All Fields] OR "gendering"[All Fields] OR "genderized"[All Fields] OR "genders"[All Fields] OR "sex"[MeSH Terms] OR "sex"[All Fields] OR "gender"[All Fields]) OR "gender identity"[All Fields] OR ("sex"[MeSH Terms] OR "sex"[All Fields])) AND ("hospitals"[MeSH Terms:noexp] OR ("delivery of health care"[MeSH Terms] OR ("delivery"[All Fields] AND "health"[All Fields] AND "care"[All	PubMed	81

Fields]) OR "delivery of health care"[All Fields] OR "healthcare"[All Fields] OR "healthcare s"[All Fields] OR "healthcares"[All Fields]) OR "health system"[All Fields] OR "health organization"[All Fields] OR "health organisation"[All Fields] OR "medical institutions"[All Fields] OR "primary healthcare"[All Fields] OR "medical colleges"[All Fields] OR "government hospitals"[All Fields] OR "medical research"[All Fields] OR "private hospital"[All Fields] OR "medical boards"[All Fields] OR "health workforce"[All Fields] OR "health r&d"[All Fields] OR "workplace"[MeSH Terms:noexp] OR "health associations"[All Fields] OR "health services"[All Fields] OR "human resources for health"[All Fields] OR "community health worker"[All Fields] OR ("nurse s"[All Fields] OR "nurses"[MeSH Terms] OR "nurses"[All Fields] OR "nurse"[All Fields] OR "nurses s"[All Fields]) OR ("midwife s"[All Fields] OR "midwifery"[MeSH Terms] OR "midwifery"[All Fields] OR "midwife"[All Fields] OR "midwifes"[All Fields] OR "midwifing"[All Fields]) OR ("physician s"[All Fields] OR "physicians"[MeSH Terms] OR "physicians"[All Fields] OR "physician"[All Fields] OR "physicians s"[All Fields]))) AND ("methods"[MeSH Terms:noexp] OR ("program"[All Fields] OR "program s"[All Fields] OR "programe"[All Fields] OR "programed"[All Fields] OR "programes"[All Fields] OR "programing"[All Fields] OR "programmability"[All Fields] OR "programmable"[All Fields] OR "programmably"[All Fields] OR "programme"[All Fields] OR "programme s"[All Fields] OR "programmed"[All Fields] OR "programmer"[All Fields] OR "programmer s"[All Fields] OR "programmers"[All Fields] OR "programmes"[All Fields] OR "programming"[All Fields] OR "programmings"[All Fields] OR "programs"[All Fields]) OR ("strategie"[All Fields] OR "strategies"[All Fields] OR "strategy"[All Fields] OR "strategy s"[All Fields]) OR ("practicability"[All Fields] OR "practicable"[All Fields] OR "practical"[All Fields] OR "practicalities"[All Fields] OR "practicality"[All Fields] OR "practically"[All Fields] OR "practicals"[All Fields] OR "practice"[All Fields] OR "practice s"[All Fields] OR "practiced"[All Fields] OR "practices"[All Fields] OR "practicing"[All Fields]) OR ("policy"[MeSH Terms] OR "policy"[All Fields] OR "policies"[All Fields] OR "policy s"[All Fields])) AND ("leadership"[MeSH Terms:noexp] OR "career mobility"[All Fields] OR "career progression"[All Fields] OR "career development"[All Fields] OR "career growth"[All Fields] OR "professional advancement"[All Fields] OR "career progress"[All Fields] OR "career path"[All Fields] OR "job progress"[All Fields] OR "key decision makers"[All Fields] OR "decision makers"[All Fields] OR "health policy makers"[All Fields] OR "health directorates"[All Fields] OR "diverse leadership"[All Fields] OR (("gender identity"[MeSH Terms] OR ("gender"[All Fields] AND "identity"[All Fields]) OR "gender identity"[All Fields] OR "gendered"[All Fields] OR "gender s"[All Fields] OR "gendering"[All Fields] OR "genderized"[All Fields] OR "genders"[All Fields] OR "sex"[MeSH Terms] OR "sex"[All Fields] OR "gender"[All Fields]) AND ("transformational"[All Fields] OR "transformative"[All Fields]) AND ("leadership"[MeSH Terms] OR "leadership"[All Fields] OR "leadership s"[All Fields] OR "leaderships"[All Fields])) OR "transformational leadership"[All Fields] OR "transformative leadership"[All Fields])) AND (2000:2022[pdat])		
((("india"[MeSH Terms] OR "india"[All Fields] OR "india s"[All Fields] OR "indias"[All Fields] OR "Uttar Pradesh"[All Fields] OR "Bihar"[All Fields] OR "Indian States"[All Fields] OR "Indian Regions"[All Fields] OR "North India"[All Fields]) NOT "North America"[All Fields]) AND (("woman*"[All Fields] OR "man"[All Fields] OR ("femal"[All Fields] OR "female"[MeSH Terms] OR "female"[All Fields] OR "females"[All Fields] OR "female s"[All Fields] OR "femals"[All Fields]) OR ("male"[MeSH Terms] OR "male"[All Fields]) OR ("gender identity"[MeSH Terms] OR ("gender"[All Fields] AND "identity"[All Fields]) OR "gender identity"[All Fields] OR "gendered"[All Fields] OR "gender s"[All Fields] OR "gendering"[All Fields] OR "genderized"[All Fields] OR "genders"[All Fields] OR "sex"[MeSH Terms] OR "sex"[All Fields] OR "gender"[All Fields]) OR "gender identity"[All Fields] OR ("sex"[MeSH Terms] OR "sex"[All Fields])) AND ("hospitals"[MeSH Terms:noexp] OR ("delivery of health care"[MeSH Terms] OR ("delivery"[All Fields] AND "health"[All Fields] AND "care"[All Fields]) OR "delivery of health care"[All Fields] OR "healthcare"[All Fields] OR "healthcare s"[All Fields] OR "healthcares"[All Fields]) OR "health system"[All Fields] OR "health organization"[All Fields] OR "health organisation"[All Fields] OR "medical institutions"[All	PubMed	190

Fields] OR "primary healthcare"[All Fields] OR "medical colleges"[All Fields] OR "government hospitals"[All Fields] OR "medical research"[All Fields] OR "private hospital"[All Fields] OR "medical boards"[All Fields] OR "health workforce"[All Fields] OR "health r&d"[All Fields] OR "workplace"[MeSH Terms:noexp] OR "health associations"[All Fields] OR "health services"[All Fields] OR "human resources for health"[All Fields] OR "community health worker"[All Fields] OR ("nurse s"[All Fields] OR "nurses"[MeSH Terms] OR "nurses"[All Fields] OR "nurse"[All Fields] OR "nurses s"[All Fields]) OR ("midwife s"[All Fields] OR "midwifery"[MeSH Terms] OR "midwifery"[All Fields] OR "midwife"[All Fields] OR "midwifes"[All Fields] OR "midwifing"[All Fields]) OR ("physician s"[All Fields] OR "physicians"[MeSH Terms] OR "physicians"[All Fields] OR "physician"[All Fields] OR "physicians s"[All Fields]))) AND ("methods"[MeSH Terms:noexp] OR ("program"[All Fields] OR "program s"[All Fields] OR "programe"[All Fields] OR "programed"[All Fields] OR "programes"[All Fields] OR "programing"[All Fields] OR "programmability"[All Fields] OR "programmable"[All Fields] OR "programmably"[All Fields] OR "programme"[All Fields] OR "programme s"[All Fields] OR "programmed"[All Fields] OR "programmer"[All Fields] OR "programmer s"[All Fields] OR "programmers"[All Fields] OR "programmes"[All Fields] OR "programming"[All Fields] OR "programmings"[All Fields] OR "programs"[All Fields]) OR ("strategie"[All Fields] OR "strategies"[All Fields] OR "strategy"[All Fields] OR "strategy s"[All Fields]) OR ("practicability"[All Fields] OR "practicable"[All Fields] OR "practical"[All Fields] OR "practicalities"[All Fields] OR "practicality"[All Fields] OR "practically"[All Fields] OR "practicals"[All Fields] OR "practice"[All Fields] OR "practice s"[All Fields] OR "practiced"[All Fields] OR "practices"[All Fields] OR "practicing"[All Fields]) OR ("policy"[MeSH Terms] OR "policy"[All Fields] OR "policies"[All Fields] OR "policy s"[All Fields])) AND ("leadership"[MeSH Terms:noexp] OR "career mobility"[All Fields] OR "career progression"[All Fields] OR "career development"[All Fields] OR "career growth"[All Fields] OR "professional advancement"[All Fields] OR "career progress"[All Fields] OR "career path"[All Fields] OR "job progress"[All Fields] OR "key decision makers"[All Fields] OR "decision makers"[All Fields] OR "health policy makers"[All Fields] OR "health directorates"[All Fields] OR "tooth attrition"[MeSH Terms:noexp] OR "retention, psychology"[MeSH Terms:noexp] OR (("career"[All Fields] OR "careers"[All Fields]) AND ("stall"[All Fields] OR "stalled"[All Fields] OR "stalling"[All Fields] OR "stallings"[All Fields] OR "stalls"[All Fields])) OR ("lack"[All Fields] AND ("promote"[All Fields] OR "promoted"[All Fields] OR "promotes"[All Fields] OR "promoting"[All Fields] OR "promotion"[All Fields] OR "promotional"[All Fields] OR "promotions"[All Fields] OR "promotive"[All Fields])) OR ("non"[All Fields] AND ("represent"[All Fields] OR "representative"[All Fields] OR "representatively"[All Fields] OR "representativeness"[All Fields] OR "representatives"[All Fields] OR "representativity"[All Fields] OR "represented"[All Fields] OR "representing"[All Fields] OR "represents"[All Fields]) AND ("leadership"[MeSH Terms] OR "leadership"[All Fields] OR "leadership s"[All Fields] OR "leaderships"[All Fields])) OR "glass ceiling"[All Fields])) AND (2000:2022[pdat])		
(((ALL=(health system)) AND ALL=(intervention OR policy)) AND ALL=(career progression)) AND ALL=(India)	Web of Science	7
((((ALL=(women)) AND ALL=(health organisation)) AND ALL=(strategy OR practice OR policy OR intervention)) AND ALL=(leadership)) AND ALL=(india)	Web of Science	15
ALL=(women AND (health organisation) AND (strategy OR practice OR policy OR intervention) AND (glass ceiling OR career stalling OR attrition)) AND ALL=(India)	Web of Science	7

((((ALL=(women)) AND ALL=(health workforce)) AND ALL=(equity OR equality OR bias OR leadership labyrinth OR occupational segregation OR enable OR prohibit)) AND ALL=(career development OR leadership OR decision makers OR career progression OR transformative leadership)) AND ALL=(India)	Web of Scien ce	3
((ALL=((health workforce OR ministry of health OR department of health) AND (equity OR equality OR bias OR leadership labyrinth OR occupational segregation))) AND ALL=(career development OR leadership OR decision makers OR career progression OR transformative leadership)) AND ALL=(India)	Web of Scien ce	132
Woman AND healthcare AND "glass ceiling" AND leadership AND India	JSTO R	9
woman OR man OR female OR male OR gender OR "gender identity" OR sex AND hospitals OR healthcare OR "health system" OR "health organization" OR "health organisation" OR "medical institutions" OR "primary healthcare" OR "medical colleges" OR "government hospitals" OR "medical research" OR "private hospital" OR "medical boards" OR "health workforce" OR "health r&d" OR workplace OR "health associations" OR "health services" OR "human resources for health" OR "community health worker" OR nurse OR midwife OR physician AND india OR "Uttar Pradesh" OR bihar OR "Indian States" OR "Indian Regions" OR "North India" not "North America" AND "glass ceiling" OR "leadership labyrinth" OR "occupational segregation" OR "vertical segregation" OR bias OR "gender bias" OR stereotype OR "gender stereotype" OR equality[OR equity OR inequality OR opportunity OR discrimination inequalities OR inequities OR inequity OR sexism AND leadership OR leaders OR " career mobility" OR "career progression" OR "career development" OR "career growth" OR "professional advancement" OR "career progress" OR "career path" OR "job progress" OR "key decision makers" OR "decision makers" OR "health policy makers" OR "health directorates" OR director AND manager OR "diverse leadership" OR "gender transformative leadership" OR "transformational leadership" OR "transformative leadership"	SCO PUS	373
woman OR man OR female OR male OR gender OR "gender identity" OR sex AND hospitals OR healthcare OR "health system" OR "health organization" OR "health organisation" OR "medical institutions" OR "primary healthcare" OR "medical colleges" OR "government hospitals" OR "medical research" OR "private hospital" OR "medical boards" OR "health workforce" OR "health r&d" OR workplace OR "health associations" OR "health services" OR "human resources for health" OR "community health worker" OR nurse OR midwife OR physician AND india OR "Uttar Pradesh" OR bihar OR "Indian States" OR "Indian Regions" OR "North India" not "North America" AND "glass ceiling" OR "leadership labyrinth" OR "occupational segregation" OR "vertical segregation" OR bias OR "gender bias" OR stereotype OR "gender stereotype" OR equality[OR equity OR inequality OR opportunity OR discrimination OR inequalities OR inequities OR inequity OR sexism or AND barrier OR inhibitor OR promote OR enable OR enabler OR prohibit AND leadership OR leaders OR "career mobility" OR "career progression" OR "career development" OR "career growth" OR "professional advancement" OR "career progress" OR "career path" OR "job progress" OR "key decision makers" OR "decision makers" OR "health policy makers" OR "health directorates" OR director AND manager OR "diverse leadership" OR "gender transformative leadership" OR "transformational leadership" OR "transformative leadership" OR attrition OR retention OR "career stalling" OR "lack of promotion" OR "non representative leadership" OR "glass ceiling"	SCO PUS	361

woman OR man OR female OR male OR gender OR "gender identity" OR sex AND hospitals OR healthcare OR "health system" OR "health organization" OR "health organisation" OR "medical institutions" OR "primary healthcare" OR "medical colleges" OR "government hospitals" OR "medical research" OR "private hospital" OR "medical boards" OR "health workforce" OR "health r&d" OR workplace OR "health associations" OR "health services" OR "human resources for health" OR "community health worker" OR nurse OR midwife OR physician AND India OR "Uttar Pradesh" OR Bihar OR "Indian States" OR "Indian Regions" OR "North India" NOT "North America" AND Intervention OR program OR strategy OR practice OR policy AND Leadership OR leaders OR "career mobility" OR "career progression" OR "career development" OR "career growth" OR "professional advancement" OR "career progress" OR "career path" OR "job progress" OR "key decision makers" OR "decision makers" OR "health policy makers" OR "health directorates" OR director OR manager OR "diverse leadership" OR "gender transformative leadership" OR "transformational leadership" OR "transformative leadership" OR attrition OR retention OR "career stalling" OR "lack of promotion" OR "non representative leadership" OR "glass ceiling"	SCO PUS	1500

**Kenya-related papers**		
**Search strategy**	**Data base**	**Res ults**
("woman*"[All Fields] OR "man"[All Fields] OR ("femal"[All Fields] OR "female"[MeSH Terms] OR "female"[All Fields] OR "females"[All Fields] OR "female s"[All Fields] OR "femals"[All Fields]) OR ("male"[MeSH Terms] OR "male"[All Fields]) OR ("gender identity"[MeSH Terms] OR ("gender"[All Fields] AND "identity"[All Fields]) OR "gender identity"[All Fields] OR "gendered"[All Fields] OR "gender s"[All Fields] OR "gendering"[All Fields] OR "genderized"[All Fields] OR "genders"[All Fields] OR "sex"[MeSH Terms] OR "sex"[All Fields] OR "gender"[All Fields]) OR "gender identity"[All Fields] OR ("sex"[MeSH Terms] OR "sex"[All Fields])) AND ("hospitals"[MeSH Terms:noexp] OR ("delivery of health care"[MeSH Terms] OR ("delivery"[All Fields] AND "health"[All Fields] AND "care"[All Fields]) OR "delivery of health care"[All Fields] OR "healthcare"[All Fields] OR "healthcare s"[All Fields] OR "healthcares"[All Fields]) OR "health system"[All Fields] OR "health organization"[All Fields] OR "health organisation"[All Fields] OR "medical institutions"[All Fields] OR "primary healthcare"[All Fields] OR "medical colleges"[All Fields] OR "government hospitals"[All Fields] OR "medical research"[All Fields] OR "private hospital"[All Fields] OR "medical boards"[All Fields] OR "health workforce"[All Fields] OR "health r&d"[All Fields] OR "workplace"[MeSH Terms:noexp] OR "health associations"[All Fields] OR "health services"[All Fields] OR "human resources for health"[All Fields] OR "community health worker"[All Fields] OR ("nurse s"[All Fields] OR "nurses"[MeSH Terms] OR "nurses"[All Fields] OR "nurse"[All Fields] OR "nurses s"[All Fields]) OR ("midwife s"[All Fields] OR "midwifery"[MeSH Terms] OR "midwifery"[All Fields] OR "midwife"[All Fields] OR "midwifes"[All Fields] OR "midwifing"[All Fields]) OR ("physician s"[All Fields] OR "physicians"[MeSH Terms] OR "physicians"[All Fields] OR "physician"[All Fields] OR "physicians s"[All Fields])) AND ("kenya"[MeSH Terms] OR "kenya"[All Fields] OR "kenya s"[All Fields] OR "Kenyan counties"[All Fields] OR (("kenya"[MeSH Terms] OR "kenya"[All Fields] OR "kenya s"[All Fields]) AND ("counties"[All Fields] OR "county"[All Fields] OR "county s"[All Fields]))) AND ("leadership"[MeSH Terms:noexp] OR "career mobility"[All Fields] OR "career progression"[All Fields] OR "career development"[All Fields] OR "career growth"[All Fields] OR "professional advancement"[All Fields] OR "career progress"[All Fields] OR "career path"[All Fields] OR "job progress"[All Fields] OR "key decision makers"[All Fields] OR "decision makers"[All Fields] OR "health policy makers"[All Fields] OR "health directorates"[All Fields] OR "diverse leadership"[All Fields] OR (("gender	PubM ed	7

identity"[MeSH Terms] OR ("gender"[All Fields] AND "identity"[All Fields]) OR "gender identity"[All Fields] OR "gendered"[All Fields] OR "gender s"[All Fields] OR "gendering"[All Fields] OR "genderized"[All Fields] OR "genders"[All Fields] OR "sex"[MeSH Terms] OR "sex"[All Fields] OR "gender"[All Fields]) AND ("transformational"[All Fields] OR "transformative"[All Fields]) AND ("leadership"[MeSH Terms] OR "leadership"[All Fields] OR "leadership s"[All Fields] OR "leaderships"[All Fields])) OR "transformational leadership"[All Fields] OR "transformative leadership"[All Fields]) AND ("glass ceiling"[All Fields] OR "leadership labyrinth"[All Fields] OR "occupational segregation"[All Fields] OR "vertical segregation"[All Fields] OR ("bias"[MeSH Terms] OR "bias"[All Fields]) OR "gender bias"[All Fields] OR "stereotyping"[MeSH Terms:noexp] OR "gender stereotype"[All Fields] OR "discrimination, psychological"[MeSH Terms:noexp] OR "sexism"[MeSH Terms:noexp])		
("woman*"[All Fields] OR "man"[All Fields] OR ("femal"[All Fields] OR "female"[MeSH Terms] OR "female"[All Fields] OR "females"[All Fields] OR "female s"[All Fields] OR "femals"[All Fields]) OR ("male"[MeSH Terms] OR "male"[All Fields]) OR ("gender identity"[MeSH Terms] OR ("gender"[All Fields] AND "identity"[All Fields]) OR "gender identity"[All Fields] OR "gendered"[All Fields] OR "gender s"[All Fields] OR "gendering"[All Fields] OR "genderized"[All Fields] OR "genders"[All Fields] OR "sex"[MeSH Terms] OR "sex"[All Fields] OR "gender"[All Fields]) OR "gender identity"[All Fields] OR ("sex"[MeSH Terms] OR "sex"[All Fields])) AND ("hospitals"[MeSH Terms:noexp] OR ("delivery of health care"[MeSH Terms] OR ("delivery"[All Fields] AND "health"[All Fields] AND "care"[All Fields]) OR "delivery of health care"[All Fields] OR "healthcare"[All Fields] OR "healthcare s"[All Fields] OR "healthcares"[All Fields]) OR "health system"[All Fields] OR "health organization"[All Fields] OR "health organisation"[All Fields] OR "medical institutions"[All Fields] OR "primary healthcare"[All Fields] OR "medical colleges"[All Fields] OR "government hospitals"[All Fields] OR "medical research"[All Fields] OR "private hospital"[All Fields] OR "medical boards"[All Fields] OR "health workforce"[All Fields] OR "health r&d"[All Fields] OR "workplace"[MeSH Terms:noexp] OR "health associations"[All Fields] OR "health services"[All Fields] OR "human resources for health"[All Fields] OR "community health worker"[All Fields] OR ("nurse s"[All Fields] OR "nurses"[MeSH Terms] OR "nurses"[All Fields] OR "nurse"[All Fields] OR "nurses s"[All Fields]) OR ("midwife s"[All Fields] OR "midwifery"[MeSH Terms] OR "midwifery"[All Fields] OR "midwife"[All Fields] OR "midwifes"[All Fields] OR "midwifing"[All Fields]) OR ("physician s"[All Fields] OR "physicians"[MeSH Terms] OR "physicians"[All Fields] OR "physician"[All Fields] OR "physicians s"[All Fields])) AND ("kenya"[MeSH Terms] OR "kenya"[All Fields] OR "kenya s"[All Fields] OR "Kenyan counties"[All Fields] OR (("kenya"[MeSH Terms] OR "kenya"[All Fields] OR "kenya s"[All Fields]) AND ("counties"[All Fields] OR "county"[All Fields] OR "county s"[All Fields]))) AND ("glass ceiling"[All Fields] OR "leadership labyrinth"[All Fields] OR "occupational segregation"[All Fields] OR "vertical segregation"[All Fields] OR ("bias"[MeSH Terms] OR "bias"[All Fields]) OR "gender bias"[All Fields] OR "stereotyping"[MeSH Terms:noexp] OR "gender stereotype"[All Fields] OR "discrimination, psychological"[MeSH Terms:noexp] OR "sexism"[MeSH Terms:noexp]) AND ("leadership"[MeSH Terms:noexp] OR "career mobility"[All Fields] OR "career progression"[All Fields] OR "career development"[All Fields] OR "career growth"[All Fields] OR "professional advancement"[All Fields] OR "career progress"[All Fields] OR "career path"[All Fields] OR "job progress"[All Fields] OR "key decision makers"[All Fields] OR "decision makers"[All Fields] OR "health policy makers"[All Fields] OR "health directorates"[All Fields] OR "tooth attrition"[MeSH Terms:noexp] OR "retention, psychology"[MeSH Terms:noexp] OR (("career"[All Fields] OR "careers"[All Fields]) AND ("stall"[All Fields] OR "stalled"[All Fields] OR "stalling"[All Fields] OR "stallings"[All Fields] OR "stalls"[All Fields])) OR ("lack"[All Fields] AND ("promote"[All Fields] OR "promoted"[All Fields] OR "promotes"[All Fields] OR "promoting"[All Fields] OR "promotion"[All Fields] OR "promotional"[All Fields] OR "promotions"[All Fields] OR "promotive"[All Fields])) OR ("non"[All Fields] AND ("represent"[All Fields] OR "representative"[All Fields] OR "representatively"[All Fields] OR "representativeness"[All Fields] OR "representatives"[All Fields] OR "representativity"[All Fields] OR "represented"[All Fields] OR "representing"[All	PubMed	7

Fields] OR "represents"[All Fields]) AND ("leadership"[MeSH Terms] OR "leadership"[All Fields] OR "leadership s"[All Fields] OR "leaderships"[All Fields])) OR "glass ceiling"[All Fields])		
(("woman*"[All Fields] OR "man"[All Fields] OR ("femal"[All Fields] OR "female"[MeSH Terms] OR "female"[All Fields] OR "females"[All Fields] OR "female s"[All Fields] OR "femals"[All Fields]) OR ("male"[MeSH Terms] OR "male"[All Fields]) OR ("gender identity"[MeSH Terms] OR ("gender"[All Fields] AND "identity"[All Fields]) OR "gender identity"[All Fields] OR "gendered"[All Fields] OR "gender s"[All Fields] OR "gendering"[All Fields] OR "genderized"[All Fields] OR "genders"[All Fields] OR "sex"[MeSH Terms] OR "sex"[All Fields] OR "gender"[All Fields]) OR "gender identity"[All Fields] OR ("sex"[MeSH Terms] OR "sex"[All Fields])) AND ("hospitals"[MeSH Terms:noexp] OR ("delivery of health care"[MeSH Terms] OR ("delivery"[All Fields] AND "health"[All Fields] AND "care"[All Fields]) OR "delivery of health care"[All Fields] OR "healthcare"[All Fields] OR "healthcare s"[All Fields] OR "healthcares"[All Fields]) OR "health system"[All Fields] OR "health organization"[All Fields] OR "health organisation"[All Fields] OR "medical institutions"[All Fields] OR "primary healthcare"[All Fields] OR "medical colleges"[All Fields] OR "government hospitals"[All Fields] OR "medical research"[All Fields] OR "private hospital"[All Fields] OR "medical boards"[All Fields] OR "health workforce"[All Fields] OR "health r&d"[All Fields] OR "workplace"[MeSH Terms:noexp] OR "health associations"[All Fields] OR "health services"[All Fields] OR "human resources for health"[All Fields] OR "community health worker"[All Fields] OR ("nurse s"[All Fields] OR "nurses"[MeSH Terms] OR "nurses"[All Fields] OR "nurse"[All Fields] OR "nurses s"[All Fields]) OR ("midwife s"[All Fields] OR "midwifery"[MeSH Terms] OR "midwifery"[All Fields] OR "midwife"[All Fields] OR "midwifes"[All Fields] OR "midwifing"[All Fields]) OR ("physician s"[All Fields] OR "physicians"[MeSH Terms] OR "physicians"[All Fields] OR "physician"[All Fields] OR "physicians s"[All Fields])) AND ("kenya"[MeSH Terms] OR "kenya"[All Fields] OR "kenya s"[All Fields] OR "Kenyan counties"[All Fields] OR (("kenya"[MeSH Terms] OR "kenya"[All Fields] OR "kenya s"[All Fields]) AND ("counties"[All Fields] OR "county"[All Fields] OR "county s"[All Fields]))) AND ("methods"[MeSH Terms:noexp] OR ("program"[All Fields] OR "program s"[All Fields] OR "programe"[All Fields] OR "programed"[All Fields] OR "programes"[All Fields] OR "programing"[All Fields] OR "programmability"[All Fields] OR "programmable"[All Fields] OR "programmably"[All Fields] OR "programme"[All Fields] OR "programme s"[All Fields] OR "programmed"[All Fields] OR "programmer"[All Fields] OR "programmer s"[All Fields] OR "programmers"[All Fields] OR "programmes"[All Fields] OR "programming"[All Fields] OR "programmings"[All Fields] OR "programs"[All Fields]) OR ("strategie"[All Fields] OR "strategies"[All Fields] OR "strategy"[All Fields] OR "strategy s"[All Fields]) OR ("practicability"[All Fields] OR "practicable"[All Fields] OR "practical"[All Fields] OR "practicalities"[All Fields] OR "practicality"[All Fields] OR "practically"[All Fields] OR "practicals"[All Fields] OR "practice"[All Fields] OR "practice s"[All Fields] OR "practiced"[All Fields] OR "practices"[All Fields] OR "practicing"[All Fields]) OR ("policy"[MeSH Terms] OR "policy"[All Fields] OR "policies"[All Fields] OR "policy s"[All Fields])) AND ("leadership"[MeSH Terms:noexp] OR "career mobility"[All Fields] OR "career progression"[All Fields] OR "career development"[All Fields] OR "career growth"[All Fields] OR "professional advancement"[All Fields] OR "career progress"[All Fields] OR "career path"[All Fields] OR "job progress"[All Fields] OR "key decision makers"[All Fields] OR "decision makers"[All Fields] OR "health policy makers"[All Fields] OR "health directorates"[All Fields] OR "diverse leadership"[All Fields] OR (("gender identity"[MeSH Terms] OR ("gender"[All Fields] AND "identity"[All Fields]) OR "gender identity"[All Fields] OR "gendered"[All Fields] OR "gender s"[All Fields] OR "gendering"[All Fields] OR "genderized"[All Fields] OR "genders"[All Fields] OR "sex"[MeSH Terms] OR "sex"[All Fields] OR "gender"[All Fields]) AND ("transformational"[All Fields] OR "transformative"[All Fields]) AND ("leadership"[MeSH Terms] OR "leadership"[All Fields] OR "leadership s"[All Fields] OR "leaderships"[All Fields])) OR "transformational leadership"[All Fields] OR "transformative leadership"[All Fields])) AND (2000:2022[pdat])	PubMed	63

(("woman*"[All Fields] OR "man"[All Fields] OR ("femal"[All Fields] OR "female"[MeSH Terms] OR "female"[All Fields] OR "females"[All Fields] OR "female s"[All Fields] OR "femals"[All Fields]) OR ("male"[MeSH Terms] OR "male"[All Fields]) OR ("gender identity"[MeSH Terms] OR ("gender"[All Fields] AND "identity"[All Fields]) OR "gender identity"[All Fields] OR "gendered"[All Fields] OR "gender s"[All Fields] OR "gendering"[All Fields] OR "genderized"[All Fields] OR "genders"[All Fields] OR "sex"[MeSH Terms] OR "sex"[All Fields] OR "gender"[All Fields]) OR "gender identity"[All Fields] OR ("sex"[MeSH Terms] OR "sex"[All Fields])) AND ("hospitals"[MeSH Terms:noexp] OR ("delivery of health care"[MeSH Terms] OR ("delivery"[All Fields] AND "health"[All Fields] AND "care"[All Fields]) OR "delivery of health care"[All Fields] OR "healthcare"[All Fields] OR "healthcare s"[All Fields] OR "healthcares"[All Fields]) OR "health system"[All Fields] OR "health organization"[All Fields] OR "health organisation"[All Fields] OR "medical institutions"[All Fields] OR "primary healthcare"[All Fields] OR "medical colleges"[All Fields] OR "government hospitals"[All Fields] OR "medical research"[All Fields] OR "private hospital"[All Fields] OR "medical boards"[All Fields] OR "health workforce"[All Fields] OR "health r&d"[All Fields] OR "workplace"[MeSH Terms:noexp] OR "health associations"[All Fields] OR "health services"[All Fields] OR "human resources for health"[All Fields] OR "community health worker"[All Fields] OR ("nurse s"[All Fields] OR "nurses"[MeSH Terms] OR "nurses"[All Fields] OR "nurse"[All Fields] OR "nurses s"[All Fields]) OR ("midwife s"[All Fields] OR "midwifery"[MeSH Terms] OR "midwifery"[All Fields] OR "midwife"[All Fields] OR "midwifes"[All Fields] OR "midwifing"[All Fields]) OR ("physician s"[All Fields] OR "physicians"[MeSH Terms] OR "physicians"[All Fields] OR "physician"[All Fields] OR "physicians s"[All Fields])) AND ("kenya"[MeSH Terms] OR "kenya"[All Fields] OR "kenya s"[All Fields] OR "Kenyan counties"[All Fields] OR (("kenya"[MeSH Terms] OR "kenya"[All Fields] OR "kenya s"[All Fields]) AND ("counties"[All Fields] OR "county"[All Fields] OR "county s"[All Fields]))) AND ("methods"[MeSH Terms:noexp] OR ("program"[All Fields] OR "program s"[All Fields] OR "programe"[All Fields] OR "programed"[All Fields] OR "programes"[All Fields] OR "programing"[All Fields] OR "programmability"[All Fields] OR "programmable"[All Fields] OR "programmably"[All Fields] OR "programme"[All Fields] OR "programme s"[All Fields] OR "programmed"[All Fields] OR "programmer"[All Fields] OR "programmer s"[All Fields] OR "programmers"[All Fields] OR "programmes"[All Fields] OR "programming"[All Fields] OR "programmings"[All Fields] OR "programs"[All Fields]) OR ("strategie"[All Fields] OR "strategies"[All Fields] OR "strategy"[All Fields] OR "strategy s"[All Fields]) OR ("practicability"[All Fields] OR "practicable"[All Fields] OR "practical"[All Fields] OR "practicalities"[All Fields] OR "practicality"[All Fields] OR "practically"[All Fields] OR "practicals"[All Fields] OR "practice"[All Fields] OR "practice s"[All Fields] OR "practiced"[All Fields] OR "practices"[All Fields] OR "practicing"[All Fields]) OR ("policy"[MeSH Terms] OR "policy"[All Fields] OR "policies"[All Fields] OR "policy s"[All Fields])) AND ("leadership"[MeSH Terms:noexp] OR "career mobility"[All Fields] OR "career progression"[All Fields] OR "career development"[All Fields] OR "career growth"[All Fields] OR "professional advancement"[All Fields] OR "career progress"[All Fields] OR "career path"[All Fields] OR "job progress"[All Fields] OR "key decision makers"[All Fields] OR "decision makers"[All Fields] OR "health policy makers"[All Fields] OR "health directorates"[All Fields] OR "tooth attrition"[MeSH Terms:noexp] OR "retention, psychology"[MeSH Terms:noexp] OR (("career"[All Fields] OR "careers"[All Fields]) AND ("stall"[All Fields] OR "stalled"[All Fields] OR "stalling"[All Fields] OR "stallings"[All Fields] OR "stalls"[All Fields])) OR ("lack"[All Fields] AND ("promote"[All Fields] OR "promoted"[All Fields] OR "promotes"[All Fields] OR "promoting"[All Fields] OR "promotion"[All Fields] OR "promotional"[All Fields] OR "promotions"[All Fields] OR "promotive"[All Fields])) OR ("non"[All Fields] AND ("represent"[All Fields] OR "representative"[All Fields] OR "representatively"[All Fields] OR "representativeness"[All Fields] OR "representatives"[All Fields] OR "representativity"[All Fields] OR "represented"[All Fields] OR "representing"[All Fields] OR "represents"[All Fields]) AND ("leadership"[MeSH Terms] OR "leadership"[All Fields] OR "leadership s"[All Fields] OR "leaderships"[All Fields])) OR "glass ceiling"[All Fields])) AND (2000:2022[pdat])	PubMed	134

(((ALL=(health system)) AND ALL=(intervention OR policy)) AND ALL=(career progression)) AND ALL=(Kenya)	Web of Scien ce	7
((((ALL=(women)) AND ALL=(health organisation)) AND ALL=(strategy OR practice OR policy OR intervention)) AND ALL=(leadership)) AND ALL=(Kenya)	Web of Scien ce	16
(ALL=(women AND (health organisation) AND (strategy OR practice OR policy OR intervention) AND (glass ceiling OR career stalling OR attrition))) AND ALL=(Kenya)	Web of Scien ce	3
((((ALL=(women)) AND ALL=(health workforce)) AND ALL=(equity OR equality OR bias OR leadership labyrinth OR occupational segregation OR enable OR prohibit)) AND ALL=(career development OR leadership OR decision makers OR career progression OR transformative leadership)) AND ALL=(Kenya)	Web of Scien ce	5
((ALL=((health workforce OR ministry of health OR department of health) AND (equity OR equality OR bias OR leadership labyrinth OR occupational segregation))) AND ALL=(career development OR leadership OR decision makers OR career progression OR transformative leadership)) AND ALL=(Kenya)	Web of Scien ce	76
Woman AND healthcare AND "glass ceiling" AND leadership AND Kenya	JSTO R	6
(woman OR man OR female OR male OR gender OR "gender identity" OR sex) AND (hospitals OR healthcare OR "health system" OR "health organization" OR "health organisation" OR "medical institutions" OR "primary healthcare" OR "medical colleges" OR "government hospitals" OR "medical research" OR "private hospital" OR "medical boards" OR "health workforce" OR "health r&d" OR workplace OR "health associations" OR "health services" OR "human resources for health" OR "community health worker" OR nurse OR midwife OR physician) AND (kenya OR "Kenyan counties" OR "Kenya counties") AND ("glass ceiling" OR "leadership labyrinth" OR "occupational segregation" OR "vertical segregation" OR bias OR "gender bias" OR stereotype OR "gender stereotype" OR equality OR equity OR inequality OR opportunity OR discrimination OR inequalities OR inequities OR inequity OR sexism) AND (leadership OR leaders OR "career mobility" OR "career progression" OR "career development" OR "career growth" OR "professional advancement" OR "career progress" OR "career path" OR "job progress" OR "key decision makers" OR "decision makers" OR "health policy makers" OR "health directorates" OR director OR manager OR "diverse leadership" OR "gender transformative leadership" OR "transformational leadership" OR "transformative leadership") AND (LIMIT-TO (PUBSTAGE , "final")) AND (LIMIT-TO (DOCTYPE , "ar")) AND (LIMIT-TO (LANGUAGE , "English"))	SCO PUS	363
woman OR man OR female OR male OR gender OR "gender identity" OR sex AND hospitals OR healthcare OR "health system" OR "health organization" OR "health organisation" OR "medical institutions" OR "primary healthcare" OR "medical colleges" OR "government hospitals" OR "medical research" OR "private hospital" OR "medical boards" OR "health workforce" OR "health r&d" OR workplace OR "health associations" OR "health services" OR "human resources for health" OR "community health worker" OR nurse OR midwife OR physician AND Kenya OR "Kenyan counties" OR "Kenya counties" AND "glass ceiling" OR "leadership labyrinth" OR "occupational segregation" OR "vertical segregation" OR bias OR "gender bias" OR stereotype OR "gender stereotype" OR equality OR equity	SCO PUS	1000

OR inequality OR opportunity OR discrimination OR inequalities OR inequities OR inequity OR sexismOR barrier OR inhibitor OR promote OR enable OR enabler OR prohibit AND Leadership OR leaders OR "career mobility" OR "career progression" OR "career development" OR "career growth" OR "professional advancement" OR "career progress" OR "career path" OR "job progress" OR "key decision makers" OR "decision makers" OR "health policy makers" OR "health directorates" OR directorOR manager OR "diverse leadership" OR "gender transformative leadership" OR "transformational leadership" OR "transformative leadership" OR attrition OR retention OR "career stalling" OR "lack of promotion" OR "non representative leadership" OR "glass ceiling"		
woman OR man OR female OR male OR gender OR "gender identity" OR sex AND hospitals OR healthcare OR "health system" OR "health organization" OR "health organisation" OR "medical institutions" OR "primary healthcare" OR "medical colleges" OR "government hospitals" OR "medical research" OR "private hospital" OR "medical boards" OR "health workforce" OR "health r&d" OR workplace OR "health associations" OR "health services" OR "human resources for health" OR "community health worker" OR nurse OR midwife OR physician AND Kenya OR "Kenyan counties" OR "Kenya counties" AND Intervention OR program OR strategy OR practice OR policy AND Leadership OR leaders OR "career mobility" OR "career progression" OR "career development" OR "career growth" OR "professional advancement" OR "career progress" OR "career path" OR "job progress" OR "key decision makers" OR "decision makers" OR "health policy makers" OR "health directorates" OR directorOR manager OR "diverse leadership" OR "gender transformative leadership" OR "transformational leadership" OR "transformative leadership" OR attrition OR retention OR "career stalling" OR "lack of promotion" OR "non representative leadership" OR "glass ceiling"	SCO PUS	1500
(woman OR women OR female OR gender) AND health AND (hospitals OR healthcare OR "health system" OR "health organization" OR "health organisation" OR "medical" OR "health workforce" OR "nurs*" OR workplace OR "health associations" OR "health services" OR "human resources for health" OR midwife OR physician) AND (“kenya”) AND (leadership OR leaders OR "career mobility" OR "career progression" OR "career development" OR "career growth" OR "professional advancement" OR "career progress" OR "career path" OR "job progress" OR "health policy makers" OR "representation” OR “participation”)	SCO PUS	310
