# Supplementary material for: Pathways to leadership: what accounts for women’s (in)equitable career paths in the health sectors in India and Kenya? A scoping review
Source: BMJ Glob Health. 2024 Jul 17;9(7):e014745. doi: 10.1136/bmjgh-2023-014745 (PMC11261739; doi:10.1136/bmjgh-2023-014745)
Supplement: Supplementary data [file bmjgh-2023-014745supp003.pdf]

BMJ Publishing Group Limited (BMJ) disclaims all liability and responsibility arising from any reliance placed on this supplemental material which has been supplied by the author(s)

Appendix 3. Data extraction tool

			Research Questions				
**Reviewer**	**Study - short name**	**ID**	**Research Question 1.1 What is the evidence for in/equality of opportunity for women's careers in the health sector in India/Kenya?**	**Research Question 1.2 What is the evidence for other social stratifiers intersecting with gender to hinder equality and inclusive leadership for women in the health sector, in India and Kenya.**	**Research Question 2.1 What are the barriers and enablers to improving equality and inclusive leadership for women in the health sector, with a focus on India and Kenya?**	**Research Question 2.2 What organisational interventions work to improve equality and inclusive leadership for women in the health sector, with a focus on India and Kenya?**	**Inclusion decision Y/N**

Study information							Methods & Framework				Location		
**Title**	**Author**	**Year**	**Abstract**	**Journal**	**URL**	**Database**	**Qual/ Quant/ Mixed methods**	**Empirical/ Conceptual**	**Descriptive/ Interventional**	**Sample size**	**Country**	**State/ County**	**Region**

Intersectional characteristics of study population															
**Gender**	**Institutional context**	**Public/ Private**	**Race**	**Caste**	**Religion**	**Gender Identity**	**Sexual Orientation**	**Economic Status**	**Education**	**Parental status**	**Professional cadre**	**Career stage**	**Ability/ disability**	**Age**	**Other Social Stratifiers**

Study findings										
**Reported measures of career pathways - by gender (Q1.1)**	**Reported measures of career pathways - by other stratifiers (Q1.2)**	**Enablers (Q2.2) - factors supporting interventions or characteristics associated with progression**	**Empirical data on the enablers (Q2.2*)**	**Barriers to improving equality and inclusive leadership for women in the health sector (Q2.1)**	**Empirical data on the barriers (Q2.1*)**	**Presence of intervention**	**Description of intervention**	**Outcome measured in the intervention study**	**Effect of intervention on the outcome**	**Comments**
