# Supplementary material for: Pathways to leadership: what accounts for women’s (in)equitable career paths in the health sectors in India and Kenya? A scoping review
Source: BMJ Glob Health. 2024 Jul 17;9(7):e014745. doi: 10.1136/bmjgh-2023-014745 (PMC11261739; doi:10.1136/bmjgh-2023-014745)
Supplement: Supplementary data [file bmjgh-2023-014745supp004.pdf]

Author reflexivity statement

Reflexivity statement regarding the paper entitled "**Pathways to leadership: what accounts for women's (in)equitable career paths in the health sectors in India and Kenya? A scoping review"**

Engagement
Has the research team engaged constructively with the reflexivity statement?
The lead author drafted this reflexivity statement and sought inputs and approval from all co-authors.

Co-development
Have the research partners co-developed the research study?
Our scoping review entitled "**Pathways to leadership: what accounts for women's (in)equitable career paths in the health sectors in India and Kenya? A scoping review**" was co-developed by a team of researchers originating from and residing in multiple countries, employed by organisations in the UK, India and Kenya. Research partners played an integral role in every stage of the project, research design, questions, tailored methodology, analysis and interpretation. The table of authors below demonstrates how our international author team (10 women, 4 men), spanned individuals from LMIC backgrounds and individuals based in LMICs, together with individuals originating from, and based in, High Income Countries. Contributors were at various career stages and the team aimed to provide learning opportunities for early career researchers to develop skills and gain experience. We took an iterative team approach to developing the research question and investigation method, the data extraction tables and the analytical framework through regular teleconference meetings. When developing the coding matrix, we discussed as a team in a participatory face-to-face workshop to decide upon the application of the social-ecological framework and its adaptation. Everyone involved was free to state their opinion and there was active discussion at every stage and every iteration in the process.

	Country of origin	Country of residence	Career stage	Involved in co-development	Extracted &/or coded data	Joined interpretation work-shop	Sex (F = female / M = male)
**GH5050 – UK-based organisation**							
Naomi M Saville (NS)	UK	Nepal	Senior	Y	Y	Y	F
Sonja Tanaka (ST)	USA	France	Mid	Y		Y	F
Lawrence Eleh (LE)	Cameroon	France	Mid	Y	Y		F
Zahra Zeinali (ZZ)	Iran	USA	Early	Y			F
Aaron Koay (AK)	Malaysia	UK	Early				M
Kent Buse (KB)	Canada	UK	Senior	Y		Y	M
Sarah Hawkes (SH)	UK	UK	Senior	Y		Y	F
**International Center for Research on Women (ICRW) Asia- India based office of ICRW**							
Radhika Uppal (RU)	India	India	Early	Y	Y	Y	F
Sapna Kedia (SK)	India	India	Mid	Y	Y	Y	F
Sucharitha Venkatesh (SV)	India	India	Early		Y		F
Ravi Verma (RV)	India	India	Senior	Y		Y	M

**African Population Health and Research Center (APHRC) – organisation based in Kenya**							
Sally Atieno Odunga (SO)	Kenya	Kenya	Early	Y	Y	Y	F
Henry Owoko Odero HO)	Kenya	Kenya	Early	Y	Y	Y	M
Sylvia Kiwuwa-Muyingo (SM)	Uganda	Kenya	Senior	Y			F

Does the study address priority research questions for the LMIC partner(s)?
Researchers based in India and Kenya expressed that the issue of inequitable pathways to leadership for women working in the health sector was a priority issue of concern in their countries which is further supported by data showing disparities in leadership roles. During discussions of the data, and from researchers' personal knowledge of their context, many examples of women being unable to fulfil their potential as leaders emerged. Team members felt strongly about this and wanted the study to contribute to understanding on this issue.

Authorship
Is there a LMIC partner who is the first or last author?
The first author (NS) originates from the UK but has been based in Nepal for the last 29 years where she lives within a Nepali family. This provides her with a deep understanding of the local context and challenges which, combined with the significant contributions from our LMIC partners, ensures that the research is well-informed by LMIC expertise and perspectives. The senior author (SH) is from UK

If not, what is the explanation?
SH was allocated senior author as she took overall responsibility for guiding the research team and was most experienced in the area. The reason for NS being allocated as first author is that, as an experienced researcher based for almost 3 decades in an LMIC, she worked as the team lead on the review process, wrote the first draft of the paper and coordinated co-author inputs.

How have LMIC early career researchers been incorporated as authors?
All early career researchers who input into the study have been given authorship and most are from LMICs (LE, ZZ, RU, SV, SO,HO and AK). Early career researchers undertook most of the data extraction, compilation of the data into the socio-ecological model coding framework and wrote the first draft of narrative about each code, so as to ensure that they got to strengthen their skills in scientific writing. The lead author then combined and adapted these narrative descriptions of the analysis and pulled the findings together.

Dissemination
How are data shared with LMIC partners to address research needs?
The data generated in the process of undertaking this review are in the form of a data extraction table which is jointly owned by all partners in the study and shared in Appendix 3. LMIC research partners have also been funded to develop dissemination and advocacy materials, host dissemination events with national networks and prepare additional peer-reviewed publications in relation to the research.

Is there open access funding to improve publication dissemination?
Yes, the paper is open access.
